# Culturing Ancient Bacteria Carrying Resistance Genes from Permafrost and Comparative Genomics with Modern Isolates

**DOI:** 10.3390/microorganisms8101522

**Published:** 2020-10-03

**Authors:** Pamela Afouda, Grégory Dubourg, Anthony Levasseur, Pierre-Edouard Fournier, Jeremy Delerce, Oleg Mediannikov, Seydina M. Diene, Daniel Nahon, Didier Bourlès, Jean-Marc Rolain, Didier Raoult

**Affiliations:** 1 Aix Marseille Université, IRD, AP-HM, MEPHI, 13005 Marseille, France; afoudapamela@yahoo.fr (P.A.); greg.dubourg@gmail.com (G.D.); anthony.levasseur@univ-amu.fr (A.L.); jeremy.delerce@univ-amu.fr (J.D.); olegusss1@gmail.com (O.M.); seydina.m.ddiene@gmail.com (S.M.D.); jean-marc.rolain@univ-amu.fr (J.-M.R.); 2 IHU Méditerranée Infection, 13005 Marseille, France; pierre-edouard.fournier@univ-amu.fr; 3 UMR VITROME, SSA, Aix-Marseille Université, IRD, AP-HM, IHU-Méditerranée-Infection, 13005 Marseille, France; 4 Aix-Marseille University, CNRS, IRD, INRAE, Coll France, UM 34 CEREGE, Technopôle de l’Environnement Arbois-Méditerranée, BP80, 13545 Aix-en-Provence, France; nahon@cerege.fr (D.N.); bourles@cerege.fr (D.B.)

**Keywords:** culturomics, Siberian permafrost, resistance genes, genomic evolution

## Abstract

Long considered to be a consequence of human antibiotics use by deduction, antibiotic resistance mechanisms appear to be in fact a much older phenomenon as antibiotic resistance genes have previously been detected from millions of year-old permafrost samples. As these specimens guarantee the viability of archaic bacteria, we herein propose to apply the culturomics approach to recover the bacterial content of a Siberian permafrost sample dated, using the in situ-produced cosmogenic nuclide chlorine36 (^36^Cl), at 2.7 million years to study the dynamics of bacterial evolution in an evolutionary perspective. As a result, we cultured and sequenced the genomes of 28 ancient bacterial species including one new species. To perform genome comparison between permafrost strains and modern isolates we selected 7 of these species (i.e., *Achromobacter insolitus, Bacillus idriensis, Brevundimonas aurantiaca, Janibacter melonis, Kocuria rhizophila, Microbacterium hydrocarbonoxydans* and *Paracoccus yeei*). We observed a high level of variability in genomic content with a percentage of shared genes in the core genomes ranging from 21.23% to 55.59%. In addition, the Single Nucleotide Polymorphism (SNP) comparison between permafrost and modern strains for the same species did not allow a dating of ancient strains based on genomic content. There were no significant differences in antibiotic resistance profiles between modern and ancient isolates of each species. Acquired resistance to antibiotics was phenotypically detected in all gram-negative bacterial species recovered from permafrost, with a significant number of genes coding for antibiotic resistance detected. Taken together, these findings confirm previously obtained data that antibiotic resistance predates humanity as most of antimicrobial agents are natural weapons used in inter-microbial conflicts within the biosphere.

## 1. Introduction

Bacterial evolution dynamics, including that of antibiotic resistance coding genes has been, to date, mostly analyzed in a deductive way from biological clock-based speculations on the one hand, and from the modern detection of current antibiotic resistance mechanisms on the other. While environmental microbial ecosystems represent a diverse and underestimated source of bacterial diversity [1], a substantial part of which is shared with human beings, their study is crucial [2,3] and provides unique data on microbial evolution [4,5,6]. Ancient environmental microorganisms have a considerable survival ability which is probably crucial in extreme cold conditions, especially permafrost, which is one of the most ancient and extreme environmental ecosystems in which microbial life has been reported [6,7,8,9,10,11,12,13,14]. Permafrost sediments contain a variety of microorganisms including among others, bacteria, archaea, green algae, fungi [6,13,14,15], but also viruses [16]. Permafrost studies have demonstrated that the viability of microbial communities can be preserved under stable conditions at low temperatures for many million years [15]. Thus, these environmental specimens represent an opportunity to identify archaic microbes, while their cultures enable the study of the dynamics of genomic evolution. To better understand the dynamics of bacterial evolution, we propose, as a primary objective of this study, to assess the genomic content of cryopreserved bacteria in permafrost by comparison with that of modern strains of the same species. In parallel, many studies on ancient permafrost have reported the presence of genes coding for resistance to several classes of antibiotics such as β-lactams, tetracyclines, aminoglycosides, glycopeptides [3,17] and to various heavy metals [18] which are very closely similar to those detected in modern clinical strains. However, as resistance to antibiotics has inconstantly been detected from permafrost samples [19,20], we also aimed in this work at evaluating the presence of resistance genes and at analyzing antibiotic resistance patterns, thereby allowing to determine the role of humanity in the apparition of antibiotic resistance.

## 2. Material and Methods

### 2.1. Specimen Collection

The permafrost specimen was collected as a part of prospecting drilling works before the construction of the “Power of Siberia” pipeline at the Unga-Baga-Olonso stream bank (59°31’49.11” N, longitude: 123°0’15.58” E and elevation: 543 m) nearby the city of Olyokminsk, Siberia. A core drilling was performed in January 2016. The core samples was collected at the bottom of the permanently frozen karst cavity at 10 m depth and examined by geologists (Figure S1) to ensure the quality of the extraction, the integrity of the carrot and the concordance of the sample with geological datation tables. The outside temperature at the moment of sampling was not higher than −20 °C.

### 2.2. Specimen Processing to Remove Possible Contaminants

After defrosting at room temperature, external layers of core sample were cleaned and removed under sterile conditions with sterile water. Redox potential and pH were measured using the benchtop pH and Ion Meters (Cyberscan 510, Singapore) and salinity with a refractometer (ATAGO CO., Ltd., Tokyo, Japan), according to the manufacturer’s instructions. Each measurement was obtained in duplicate.

### 2.3. Permafrost Dating

After its mineralogical analysis (Figure S2), the permafrost sample was dated using the determination of its in situ produced cosmogenic nuclide ^36^Cl concentration [21,22]. The measurements were carried out using the accelerator mass spectrometry (AMS) technique at the French AMS national facility ASTER (CEREGE, Aix en Provence, France) (Supplementary Data).

### 2.4. Culturomics

The culturomics approach was conducted by combining two inoculation methods, 7 culture media, and 3 and incubation temperatures and atmospheres, respectively (Table S4). As a result, we applied a total of 84 culture condition combinations to the permafrost specimen (Supplementary Material). The culture media used were (5% sheep blood-enriched Columbia agar (COS) (bioMérieux, Marcy l’Etoile, France), R-Medium [23], 2 halophilic culture media [24] and 3 oligotrophic culture media that were optimized according to permafrost physicochemical properties (Supplementary Material). Grown colonies were subjected to Matrix-Assisted Laser Desorption/Ionization- time-of-flight mass spectrometry MALDI-TOF MS analysis for identification (Supplementary Material). Briefly, the acquired mass spectrum was compared with those present in a custom library containing manufacturer and personal spectra from 7463 species. An isolate was considered as correctly identified at the genus and at species levels when the identification score was ≥1.7 and ≥2, respectively. Unidentified colonies were subjected to 16S rRNA gene amplification and sequencing to achieve species identification (Supplementary Material).

### 2.5. Genomic Study

#### 2.5.1. Choice of Strains and Modern Genomes

Among strains isolated from the studied sample, we chose 7 strains according to the two following criteria (i) bacterial species not isolated in other studies of our culturomics laboratory prior to this study, thus reducing thus the risk of being a contaminant, and (ii) bacterial species for which at least 3 other different strains isolated in samples other than permafrost or 3 distinct genomes were available in a collection or database.

All 7 bacterial strains were genome sequenced. In order to compare these permafrost isolates with modern strains of the same species, we have selected and downloaded for each of them 3 modern genomes available in the NCBI genome database (https://www.ncbi.nlm.nih.gov/genome). For species for which at least 3 modern genomes were not available, we collected strains from our strain collection (i.e., CSUR: Collection de Souches de l’Unité des Rickettsies, Marseille, France) and sequenced their genomes. Genomes taken from NCBI and sequenced strains are listed in Table S1.

#### 2.5.2. DNA Extraction and Genome Sequencing

Genomic DNAs (gDNAs) of all bacterial strains sequenced were extracted in two steps: a mechanical treatment was first performed using acid washed glass beads (G4649-500g) (Sigma-Aldrich, Lyon, France) and a FastPrep BIO 101 instrument (Qbiogene, Strasbourg, France) at maximum speed (6.5 m/s) for 90 s. Then, after a 2–2.5 h lysozyme incubation at 37 °C, DNA was extracted on the EZ1 biorobot with the EZ1 DNA Tissue kit (Qiagen). The elution volume was 50 µL. Each gDNA was quantified by a Qubit assay with the high sensitivity kit (Life technologies, Carlsbad, CA, USA).

The strategy used for sequencing genomic DNAs varied from one bacterial strain to another. Four strategies were used: (i) one run with the Paired-End strategy using the MiSeq technology (Illumina, San Diego, CA, USA); (ii) two successive runs with the Paired-End Strategy using the MiSeq technology (Illumina); (iii) one run with the Paired-End Strategy using the MiSeq technology (Illumina) + one MinION sequencing run (Oxford Nanopore Technologies Ltd., Oxford, UK); (iv) one run with the Mate-Pair Strategy (Illumina) + 1 MinION sequencing run (Oxford Nanopore Technologies Ltd.). Only one of these four sequencing strategy was used for each of the studied strains (Table S1).

#### 2.5.3. Genome Assembly

The genomes were assembled using SPADES v. 3.10.1 with default parameters [23]. Contigs smaller than 500 nucleotides or with a coverage lower than 1/4 of the mean of coverage from contigs in the N50 were removed.

#### 2.5.4. Genome Annotation and Pan-Genome

We annotated assembled genomes using Prokka v. 1.13 [25] via the Galaxy platform (https://usegalaxy.eu/root?tool_id=toolshed.g2.bx.psu.edu/repos/crs4/prokka/prokka/1.13+galaxy0) selecting the parameters: “annotation in gff3 format, containing both sequences and annotations”, “protein FASTA file of the translated CDS sequences” and “nucleotide FASTA file of all the annotated sequences, not just CDS”. Then, gff3 files generated by Prokka were used to estimate the pan-genome (Galaxy v. 3.10.2) considering 80% as minimum percentage identity for blastp.

#### 2.5.5. Phylogeny and Ancestral Single Nucleotide Polymorphism (SNP)

We generated trees using the Maximum likelihood method and Mega X [26]. We obtained the ancestral SNP possibility with Mega X and the Parsimony method [27]. Then, a python script transformed csv result from MEGA into a FASTA file. uncertain ancestral SNPs were changed to the correct degenerated nucleotide according to the IUPAC code. A python script was created to calculate the number and type of difference between each pair of sequences based on a strict difference level.

### 2.6. Antibiotic Resistance

The antibiotic resistance of permafrost and modern strains was phenotypically assessed by the disk diffusion method (Supplementary Material) and by genome sequences comparison using the ARG-ANNOT [28], CARD (card.mcmaster.ca) and Resfinder (https://cge.cbs.dtu.dk/services/ResFinder/) databases. Susceptibility to β-lactams, aminoglycosides, rifamycins, fosfomycin, sulfamides, fluoroquinolones, tetracyclines were studied for all bacterial species. In addition, the susceptibility to colistin was only studied for gram-negative species while susceptibility to macrolides-lincosamides- streptogramins, fucidic acid and oxazolidinones was only assessed for gram-positive species.

## 3. Results

### 3.1. Permafrost Properties

The mineralogical analysis of the studied permafrost specimen showed a predominance of dolomite (53.9%) (Figure S2). The age of the permafrost sample determined by the accumulated ^36^Cl concentration was estimated at 2.7 ± 0.4 million years (Supplementary Data).

### 3.2. Microbiology

#### Microbial Culturomics

A total of 28 species were isolated using the culturomics approach (Table 1), one of which being a new species discovered as part of this study named *Bacillus massiliglaciei* strain Marseille-P2600^T^ (= CSUR P2600 = DSM 102861) and recently described [29]. All 28 bacterial species were oxygen-tolerant and belonged in great majority to the Firmicutes (39.29%) and Proteobacteria (32.14%) and in a smaller proportion to the Actinobacteria (25%) and Bacteroidetes (3.57%). Some species had previously been found in the environment (Table S2), whereas others had been found in humans.

### 3.3. Genomes

Out of the 28 species recovered from the permafrost specimen, seven were selected for genome comparison (Table S1).

**Figure 1:**
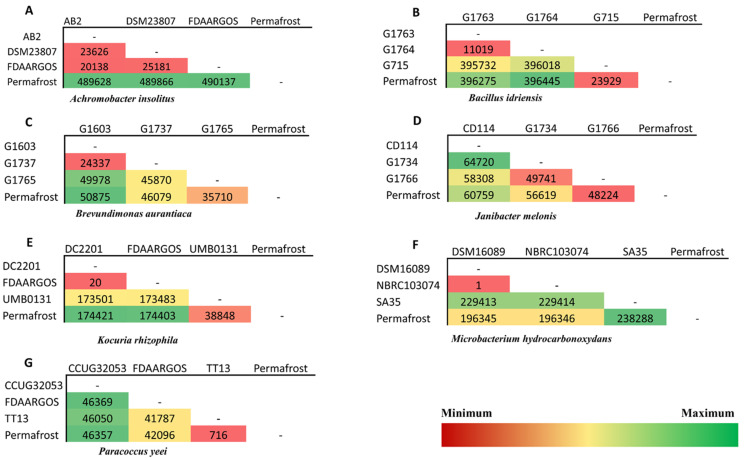
Pairwise comparisons of the number of different Single Nucleotide Polymorphisms (SNPs) between strains of the same species. Green to red shows the lowest to highest SNPs. The column named “Permafrost” represents bacterial strains isolated from permafrost. The others represent the modern non-permafrost strains. *Achromobacter insolitus* (**A**), *Bacillus idriensis* (**B**), *Brevundimonas aurantiaca* (**C**), *Janibacter melonis* (**D**), *Kocuria rhizophila* (**E**), *Microbacterium hydrocarbonoxydans* (**F**) and *Paracoccus yeei* (**G**).

Sequence variations were detected between permafrost isolates and modern strains, but also at the core genome level of species (Figure S3). The pairwise comparisons of the numbers of SNPs between strains of the same species demonstrated that permafrost strains do not systematically display the highest proportion of SNPs (Figure 1, Figure S4, Table S3 and Supplementary results).

Phylogenetic trees constructed on the basis of SNP proportions showed that the evolutionary distances of sequences from permafrost strains compared to the ancestral sequences differ from one bacterial species to another (Figure S5A–G and Figure S6A–G). However, the highest evolutionary distance with respect to the ancestral sequence was always with that that of the permafrost (Figure S4, Figure S6A–G, Figure S7) for the seven species studied. Taken together, although these data suggested that permafrost strains and modern isolates differ, it did not seem possible to identify, within a given species, the strain of archaic origin and those that are modern on a scale of 2 million years.

### 3.4. Antibiotic Resistance

#### 3.4.1. Phenotype of Resistance

**Figure 2:**
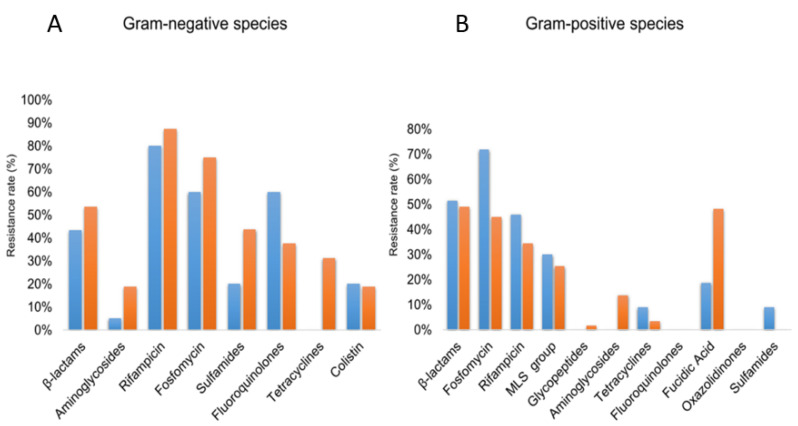
Antibiotic resistance rates among permafrost and modern strains. Only species for which both permafrost and modern strains were available for antibiotic susceptibility testing were included. (**A**) represents results from 10 gram-negative species (including 10 permafrost strains and 16 modern strains. (**B**) represents the results from 11 gram-positive bacterial species (11 permafrost strains and 29 modern strains).

By analyzing the resistance phenotypes of permafrost and modern strains, we demonstrate that all of the 10 isolated gram-negative bacteria cultured displayed at least one resistance mechanism that is not intrinsic within the bacterial species. All *Achromobacter* sp. strains displayed acquired resistance to fluoroquinolones when compared to their modern isolates. In contrast, no acquired resistance to tetracyclines was observed for the permafrost strains, as demonstrated by the doxycycline susceptibility (Figure 2A,B, Table S5A).

Regarding gram-positive strains (Table S5B), no resistance to linezolid and fluoroquinolones was observed in permafrost isolates. However, permafrost strains of *S. epidermidis* and *S. capitis* were resistant to methicillin. Finally, one isolate *Paenibacillus provencensis* isolate was found to be resistant to all tested beta-lactams when compared to modern isolates. For fosfomycin, rifamycins, macrolides and fusidic acid classes similar susceptibility profiles were observed for ancient and modern isolates (Figure 2B).

#### 3.4.2. Resistome

In total, permafrost strains harbor genes coding for resistance to 20 different antibiotic families (Figure 2; Table S6). Depending on strains, the number of antibiotic resistance genes ranged from 2 to 72. Resistance genes were mostly identified in *Enterobacter cloacae*, *Pantoea septica*, *Pantoea massiliensis*, *Achromobacter insolitus*, *Achromobacter spanius*, *Achromobacter pulmonis*, *Acinetobacter baumannii*, and *Janibacter melonis* strains (72, 69, 59, 51, 47, 43, 32 and 21 antibiotic resistance genes, respectively). The Multi-Drug Efflux class was the most predominant among antibiotic resistance genes and was detected in all strains except *Kocuria rhizophila*, *Paracoccus yeei* and *Paenisporosarcina quisquiliarum* (Table 2; Figure S8). The most prevalent genes were represented by: *AGly*, *Bla*, *Flq*, *rpoB2*, *EF-Tu*, n*ovA*, *parY* and *sav1866* (Table S6).

## 4. Discussion

Herein, we cultivated bacteria from 2.7 million-year-old Siberian permafrost sample and evaluated if it was possible to perform a molecular dating when compared to modern strains of the same species.

First of all, this work pointed out that permafrost allows the preservation of ancient prokaryotes, as 28 bacterial species could be recovered from the specimen, including one new bacterial species (i.e., *Bacillus massiliglaciei*). Several of the species cultured in the present study had already been isolated from similar specimens, including *Microbacterium, Bacillus, Paenibacillus, Kocuria and Staphylococcus* species [19]. Interestingly, using minimal medium combined with incubation at room temperature enabled to capture the maximal diversity compared to the other culture conditions used. We then selected seven bacterial species for the genome comparison. These species had not been isolated in our culturomics laboratory prior to this study, and we thus believe that these strains are not contaminants.

The comparison of the genomes sequenced from permafrost isolates and those from modern strains of the same species did not allow a molecular dating of ancient microbes. These data suggest that the molecular clock for bacteria is not reliable at such short intervals (i.e., 2.7 million years). This observation supports the idea that one of the accepted definitions of bacterial species is a 1.3% in 16S rRNA gene sequence that represents a temporal divergence of 50 million years. We assume that the design of this study implies that the modern strains are the direct descendants of the ancient strains of the same species. By neglecting the phylogenetic difference within the same species, this could constitute a limitation of this work.

The antibiotic susceptibility testing performed on permafrost isolates suggests that ancient microbes have also acquired resistance to antibiotics. Indeed, the resistance phenotypes observed do not significantly differ between the ancient and the modern isolates. As a matter of fact, 10 out of the 10 analyzable gram-negative bacteria recovered from the permafrost specimen have acquired resistance to at least one of the tested antibiotics. In parallel, the resistome analysis also highlighted a significant number of resistance genes, in particular in human pathogens such as *Pantoea septica* or *Pantoea massiliensis* (i.e., 69 and 59 genes detected, respectively) and, most importantly, *Enterobacter cloacae* for which 72 resistance genes were found. As it has been previously suggested, these data strongly support that antibiotic resistance predates by far the human use of antibiotics, which dates from only 70 years old. This is not surprising as most antibiotics are natural weapons used in inter-microbial conflicts and antibiotic resistance thereby predates the appearance of multicellular organisms [30]. The only exception to these observations may be the use of antibiotics in animal husbandry or agriculture for which the selection pressure is several levels of magnitude greater than that due to the prescription of antimicrobials in human medicine. This is probably reflected in this work by the absence of resistance to tetracyclines—an antibiotic class widely used for this purpose—among the gram-negative bacteria isolated as a part of this study. These results are in line with the low proportion of tetracycline-resistant isolates recovered from permafrost sediments previously reported [3]. Interestingly, no acquired resistance to oxazolidinones—which is a totally synthetic antibiotic class—was observed within permafrost isolates.

The bacterial diversity obtained from the permafrost sample analyzed herein is similar to that retrieved from specimen collected from an ice wedge collected also in Central Yakutia [30]. The authors found that most of the isolates were susceptible to usual antibiotics, except several strains resistant to BenzylPenicillin. However, it was shown that among bacteria isolated from Canadian high Arctic permafrost, genes coding for resistance against aminoglycosides, β-lactams and tetracyclines were detected. Of the genera isolated, *Bacillus* spp. From the permafrost and *Stenotrophomonas* spp. from the active layer were particularly associated with resistance to these 3 families [31]. Several of the identified resistance mechanisms were similar to those found in modern isolates. Finally, the present culture of resistant coliforms (i.e., *E. cloacae*) and coagulase-negative staphylococci from permafrost is in line with the findings of previous studies [31]. We acknowledge that in situ cultivation, which is efficient to capture a maximal bacterial diversity regarding environmental bacteria, could not be performed considering the specimen studied in this work.

We believe that this study may contribute to a better understanding of nature as well as the origin of antibiotic resistance through the study of archaic microbes. In a similar fashion, recent studies dedicated to the environmental antibiotic resistance in geographic areas poorly studied so far [32] suggest that the prevalence of antibiotic resistance is not directly related to the human use of antibiotics.

## 5. Conclusions

By cultivating and genome sequencing 28 bacterial species from a 2.7 million-year-old permafrost specimen, we were not able to date the ancient strains based on their genomic content. These findings suggest that the molecular clock for bacteria is not comparable with that of vertebrates. The significant number of genes coding for antibiotic resistance found and the antibiotic susceptibility testing results from ancient strains comfort in fact that acquired antibiotic resistance predates humanity.

## Figures and Tables

**Table 1:** Microarray showing bacterial species according to culture media and conditions used for their isolation (atmosphere, pH and salinity included). Green = positive culture; Red = negative culture. Cos: Columbia with 5% sheep blood agar.

Nr.	Bacterial Species	Minimal Medium	Room Temperature		Minimal Medium	30°C	R-Medium			
			COS	R-Medium		COS		CSUR Number	% GC Content	Genome Size (bp)
1	*Achromobacter denitrificans*							CSURP2856	64.21	3,568,352
2	*Achromobacter insolitus*							CSURP2857	65.60	3,505,659
3	*Achromobacter pulmonis*							CSURP5020	65.27	6,225,599
4	*Achromobacter spanius*							CSURP2943	62.98	3,795,947
5	*Acinetobacter baumannii*							CSURP2941	39.4	3,547,553
6	*Agrococcus baldri*							CSURP2731	71.77	3,021,020
7	*Bacillus idriensis*							CSURP2855	38.67	5,097,893
8	*Bacillus massiliglaciei*							CSURP2600	43.77	4,145,500
9	*Bacillus megaterium*							CSURP2736	38.30	4,914,860
10	*Bacillus simplex*							CSURP2675	41.60	3,985,190
11	*Brevundimonas aurantiaca*							CSURP3291/CSURP3513	66.09	3,046,902
12	*Enterobacter cloacae*							CSURP2676	55.3	4,119,770
13	*Janibacter melonis*							CSURP2733	70.46	2,989,541
14	*Kocuria rhizophila*							CSURP2672	70.7	2,175,710
15	*Microbacterium hydrocarbonoxydans*							CSURP2596	59.87	1,927,472
16	*Micrococcus luteus*							CSURP2671	73.5	2,169,972
17	*Paenibacillus provencensis*							CSURP2737	45.98	5,620,058
18	*Paenibacillus urinalis*							CSURP2673/CSURP3512	47.2	5,466,760
19	*Pantoea massiliensis*							CSURP5021	54.85	4,839,004
20	*Pantoea septica*							CSURP2571	58.76	3,942,569
21	*Paracoccus yeei*							CSURP2668	68.1	2,979,396
22	*Pedobacter quisquiliarum*							CSURP2597	42.6	520,513
23	*Planomicrobium glaciei*							CSURP2599	35	4,176,240
24	*Sphingomonas paucimobilis*							CSURP3292/CSURP3510	55.68	2,442,583
25	*Staphylococcus capitis*							CSURP5018	33.3	3,786,748
26	*Staphylococcus epidermidis*							CSURP3290/CSURP3514	32.3	2,318,162
27	*Staphylococcus pasteuri*							CSURP2670	37.35	2,414,997
28	*Staphylococcus saprophyticus*							CSURP2669	32.9	2,291,952

COS: Columbia agar + 5% sheep blood; CSUR: collection de souches de l’Unité des Rickettsies.

**Table 2:** Resistome of permafrost strains: BlastP analysis against ARG-ANNOT, CARD and Resfinder Databases. Different colors mean values refer to the number of antibiotic resistance genes identified by BlastP against three antibiotic resistance gene databases.

	Nbr of Contigs	Genome Size (Mb)	%GC content	Ac	Ag	Bct	BL	C	E	FF	FA	GP	ML	Mi	MDE	Mup	Nov	Ph	Pol	Q	Rif	Stg	Sul
***Achromobacter insolitus G1433***	1	6.5	65.6	3	0	0	0	0	3	0	0	0	1	0	40	0	1	0	0	2	0	0	0
***Achromobacter pulmonis G1574***	94	6.23	65.27	2	1	0	1	0	2	0	0	0	1	0	30	0	3	0	1	1	0	0	0
***Achromobacter spanius***	98	6.47	62.98	2	0	0	0	0	4	0	0	0	1	0	37	0	1	0	0	1	0	0	0
***Acinetobacter baumannii***	1	4.33	39.4	0	1	0	5	0	2	0	0	0	0	0	19	0	0	2	0	0	0	0	2
***Agroccocus massilioglaciei G1167***	3	3.02	71.77	2	0	0	0	3	1	0	0	0	0	0	1	1	6		0	1	0	0	0
***Bacillus idriensis G1436***	46	5.1	38.67	3	0	0	0	1	1	0	0	0	1	0	3	0	0	1	0	0	2	0	1
***Bacillus massilioglaciei***	19	4.14	43.77	0	0	0	0	1	1	0	0	0	1	0	3	0	0	0	0	0	0	0	0
***Bacillus megaterium G1443***	1	5.34	38.3	0	0	0	1	0	1	1	0	1	2	0	3	0	0	0	0	1	1	0	0
***Bacillus simplex G1422***	1	5.34	41.6	0	0	0	1	0	1	0	0	0	3	0	6	0	0	1	0		2	0	0
***Brevundimonas aurantiaca G1452***	94	3.12	66.09	2	0	0	0	1	2	0	0	0	1	0	4	0	1	0	0	0	0	0	0
***Enterobacter cloacae***	1	5.32	55.3	0	1	1	8	1	3	2	0	0	1	1	33	0	0	0	6	14	0	0	0
***Janibacter melonis***	7	3.2	70.46	2	0	0	0	1	1	0	0	0	2		4	1	6	0	1		2	0	0
***Kocuria rhizophila G1424***	1	2.7	70.7	2	0	0	0	2	1	0	0	0	0	0	0	1	1	0	0	0	0	0	0
***Microbacterium hydrocarbonoxydans G1438***	10	3.95	59.87	2	0	0	0	1	1	0	0	3	1	0	2	3	3	0	0	0	1	0	0
***Micrococcus luteus G1425***	1	2.5	73.5	2	0	0	0	1	1	0	0	0	2	0	1	1	2	0	0	0	0	0	0
***Paenibacillus provencensis G1439***	20	5.62	45.98	2	0	0	0	1	1	0	0	2	2	0	1	0	0	0	0	0	0	0	0
***Paenibacillus urinalis G1453***	3	5.47	47.2	1	0	0	0	1	1	0	0	2	2	0	1	0	0	0	0	0	0	0	0
***Pantoea massiliensis G1572***	65	4.83	54.85	1	0	1	8	1	1	1	0	0	1	1	32	0		1	5	3	0	2	0
***Pantoea septica G1442***	37	4.55	58.76	2	1	1	6	1	2	1	0	0	3	1	35	0	2	1	6	4	1	1	0
***Paracoccus yeei G1426***	1	3.62	68.1	0	0	0	0	0	0	0	0	0	0	0	0	0	0	0	0	0	0	0	0
***Pedobacter cryoconitis***	1	5.95	42.6	0	0	0	0	0	1	0	0	0	0	0	0	0	0	0	0	0	0	0	0
***Planomicrobium glaciei G1601***	35	4.18	35	0	1	0	0	2	1	0	0	0	1	0	0	0	0	0	0	0	2	0	0
***Sphingomonas paucimobilis G1448***	84	4.33	55.68	2	1	0	0	1	1	0	0	0	0	0	8	0	1	0	0	1	0	0	0
***Staphylococcus saprophyticus G1441***	1	2.52	33.3	0	1	0	0	2	0	1	1	0	0	0	3	0	0	1	0	4	0	0	2
***Staphylococcus epidermidis***	1	2.5	32.3	0	1	0	1	0	1	0	0	0	0	0	3	0	0	1	0	4	0	0	2
***Staphylococcus capitis G1573***	11	2.42	37.35	0	1	0	1	0	1	1	0	0	0	0	3	0	0	1	0	5	0	0	2
***Staphylococcus pasteuri G1440***	1	2.56	32.9	0	1	0	0	1	1	0	0	0	0	0	3	0	0	1	0	4	0	0	1

AC: aminocoumarin; AG: aminoglycosides; Bct: bacitracin; BL: Beta-lactams; C: cyclines; E: Elfamycin; FF: fosfomycin; FA: fusidic acid; GP: glycopeptides; ML: macrolides; Mi: microcin; MDE: multi-drug efflux; Mup: mupirocin; Nov: novobiocin; Ph: phenicols; Pol: polymyxins; Q: quinolones; Rif: rifampicin; Stg: streptogramins; Sul: sulfamids.

## Data Availability

Genomes sequences from the 7 permafrost strains used for genomic comparison were deposited in EBI under accession number PRJEB40293.

## Supplementary Materials

The following are available online at https://www.mdpi.com/2076-2607/8/10/1522/s1, Figure S1: Illustration of the permafrost collection process, Figure S2: Mineralogical analysis of the permafrost sample, Figure S3: Pan-genomes representing four strains of seven bacterial species: *Achromobacter insolitus* (A), *Bacillus idriensis* (B), *Brevundimonas aurantiaca* (C), *Janibacter melonis* (D), *Kocuria rhizophila* (E), *Microbacterium hydrocarbonoxydans* (F) and *Paracoccus yeei* (G), Figure S4: Evolutionary distances of bacterial sequences relative to each other and relative to their ancestral sequences, Figure S5: Phylogenetic trees based on the SNP core genomes highlighting the position of permafrost species compared to their modern equivalents and their ancestral sequences, Phylogenetic trees based on the SNP core genomes highlighting the position of strains of the same species compared to their ancestral sequences, Figure S7: Phylogenetic trees based on rpoB protein sequences of seven strains isolated from permafrost (*Achromobacter insolitus, Bacillus idriensis, Brevundimonas aurantiaca, Janibacter melonis, Kocuria rhizophila, Microbacterium hydrocarbonoxydans, Paracoccus yeei*) and three of the modern strains for each species, Figure S8: Resistome of permafrost strains genome: BlastP analysis against ARG-ANNOT, CARD, and Resfinder Databases, Table S1: Bacterial strains used for genomic comparisons, Table S2: Habitat of bacterial species isolated from permafrost, Table S3: Number of single-nucleotide polymorphisms in the core genome in the same species strains’: AC versus CG, Table S4: Culture conditions of Siberian Permafrost sample, Table S5A: Antibiotic susceptibility testing results from gram-negative permafrost’s strains and from their contemporary isolates available in the CSUR, Table S5B: Antibiotic susceptibility testing results from gram-positive permafrost’s strains and from their contemporary isolates available in the CSUR, Table S6: Details of the genes coding for resistance identified from the 28 genomes of permafrost strains.

## Abbreviations

CSUR	Collection de Souches de l’Unité des Rickettsies
DSMZ	Deutsche Sammlung von Mikroorganismen und Zellkulturen
MALDI-TOF MS	Matrix-assisted laser-desorption/ionization time-of-flight mass spectrometry
SNP	Single nucleotide polymorphism

## References

[1] Curtis T.P., Sloan W.T., Scannell J.W. (2002). Estimating prokaryotic diversity and its limits. Proc. Natl. Acad. Sci. USA.

[2] D’Costa V.M., McGrann K.M., Hughes D.W., Wright G.D. (2006). Sampling the antibiotic resistome. Science.

[3] Mindlin S.Z., Soina V.S., Petrova M.A., Gorlenko Z.M. (2008). Isolation of antibiotic resistance bacterial strains from Eastern Siberia permafrost sediments. Russ. J. Genet..

[4] Wayne R.K., Leonard J.A., Cooper A. (1999). Full of Sound and Fury: History of Ancient DNA. Annu. Rev. Ecol. Syst..

[5] Cano R.J., Tiefenbrunner F., Ubaldi M., Cueto C.D., Luciani S., Cox T., Orkand P., Künzel K.H., Rollo F. (2000). Sequence analysis of bacterial DNA in the colon and stomach of the Tyrolean Iceman. Am. J. Phys. Anthropol..

[6] Steven B., Léveillé R., Pollard W.H., Whyte L.G. (2006). Microbial ecology and biodiversity in permafrost. Extremophiles.

[7] Panikov N.S., Sizova M.V. (2007). Growth kinetics of microorganisms isolated from Alaskan soil and permafrost in solid media frozen down to -35 degrees C. FEMS Microbiol. Ecol..

[8] Hinsa-Leasure S.M., Bhavaraju L., Rodrigues J.L.M., Bakermans C., Gilichinsky D.A., Tiedje J.M. (2010). Characterization of a bacterial community from a Northeast Siberian seacoast permafrost sample. FEMS Microbiol. Ecol..

[9] Dmitriev V.V., Suzina N.E., Rusakova T.G., Gilichinskii D.A., Duda V.I. (2001). Ultrastructural Characteristics of Natural Forms of Microorganisms Isolated from Permafrost Grounds of Eastern Siberia by the Method of Low-Temperature Fractionation. Dokl. Biol. Sci..

[10] Frey B., Rime T., Phillips M., Stierli B., Hajdas I., Widmer F., Hartmann M. (2016). Microbial diversity in European alpine permafrost and active layers. FEMS Microbiol. Ecol..

[11] Hu W., Zhang Q., Li D., Cheng G., Mu J., Wu Q., Niu F., An L., Feng H. (2014). Diversity and community structure of fungi through a permafrost core profile from the Qinghai-Tibet Plateau of China. J. Basic Microbiol..

[12] Graham D.E., Wallenstein M.D., Vishnivetskaya T.A., Waldrop M.P., Phelps T.J., Pfiffner S.M., Onstott T.C., Whyte L.G., Rivkina E.M., Gilichinsky D.A. (2012). Microbes in thawing permafrost: The unknown variable in the climate change equation. ISME J..

[13] Zhang D.-C., Brouchkov A., Griva G., Schinner F., Margesin R. (2013). Isolation and characterization of bacteria from ancient siberian permafrost sediment. Biology.

[14] Steven B., Briggs G., McKay C.P., Pollard W.H., Greer C.W., Whyte L.G. (2007). Characterization of the microbial diversity in a permafrost sample from the Canadian high Arctic using culture-dependent and culture-independent methods. FEMS Microbiol. Ecol..

[15] Gilichinsky D.A., Vorobyova E.A., Erokhina L.G., Fyordorov-Davydov D.G., Chaikovskaya N.R., Fyordorov-Dayvdov D.G. (1992). Long-term preservation of microbial ecosystems in permafrost. Adv. Space Res..

[16] Legendre M., Lartigue A., Bertaux L., Jeudy S., Bartoli J., Lescot M., Alempic J.-M., Ramus C., Bruley C., Labadie K. (2015). In-depth study of Mollivirus sibericum, a new 30,000-y-old giant virus infecting Acanthamoeba. Proc. Natl. Acad. Sci. USA.

[17] D’Costa V.M., King C.E., Kalan L., Morar M., Sung W.W.L., Schwarz C., Froese D., Zazula G., Calmels F., Debruyne R. (2011). Antibiotic resistance is ancient. Nature.

[18] Mindlin S., Petrenko A., Kurakov A., Beletsky A., Mardanov A., Petrova M. (2016). Resistance of Permafrost and Modern Acinetobacter lwoffii Strains to Heavy Metals and Arsenic Revealed by Genome Analysis. BioMed Res. Int..

[19] Filippova S., Surgucheva N., Kolganova T., Cherbunina M.Y., Brushkov A., Mulyukin A., Gal’chenko V. (2019). Isolation and Identification of Bacteria from an Ice Wedge of the Mamontova Gora Glacial Complex (Central Yakutia). Biol. Bull..

[20] Perron G.G., Whyte L., Turnbaugh P.J., Goordial J., Hanage W.P., Dantas G., Desai M.M. (2015). Functional characterization of bacteria isolated from ancient arctic soil exposes diverse resistance mechanisms to modern antibiotics. PLoS ONE.

[21] Gilichinsky D.A., Nolte E., Basilyan A.E., Beer J., Blinov A.V., Lazarev V.E., Kholodov A.L., Meyer H., Nikolskiy P.A., Schirrmeister L. (2007). Dating of syngenetic ice wedges in permafrost with 36Cl. Quat. Sci. Rev..

[22] Tikhomirov D.A., Blinov A.V. (2009). Cosmogenic 36Cl as a tool for dating permafrost ice. Bull. Russ. Acad. Sci. Phys..

[23] Dione N., Khelaifia S., La Scola B., Lagier J.C., Raoult D. (2016). A quasi-universal medium to break the aerobic/anaerobic bacterial culture dichotomy in clinical microbiology. Clin. Microbiol. Infect..

[24] Seck E.H., Sankar S.A., Khelaifia S., Croce O., Robert C., Couderc C., Di Pinto F., Sokhna C., Fournier P.-E., Raoult D. (2016). Noncontiguous finished genome sequence and description of Planococcus massiliensis sp. nov., a moderately halophilic bacterium isolated from the human gut. New Microbes New Infect..

[25] Seemann T. (2014). Prokka: Rapid prokaryotic genome annotation. Bioinforma. Oxf. Engl..

[26] Kumar S., Stecher G., Li M., Knyaz C., Tamura K. (2018). MEGA X: Molecular Evolutionary Genetics Analysis across Computing Platforms. Mol. Biol. Evol..

[27] Swofford D.L. (2001). PAUP*: Phylogenetic Analysis Using Parsimony (and Other Methods) 4.0.b5.

[28] Gupta S.K., Padmanabhan B.R., Diene S.M., Lopez-Rojas R., Kempf M., Landraud L., Rolain J.-M. (2014). ARG-ANNOT, a new bioinformatic tool to discover antibiotic resistance genes in bacterial genomes. Antimicrob. Agents Chemother..

[29] Cadoret F., Alou M.T., Afouda P., Traore I.S., Bréchard L., Michelle C., Di Pinto F., Andrieu C., Delerce J., Levasseur A. (2017). Noncontiguous finished genome sequences and description of Bacillus massiliglaciei, Bacillus mediterraneensis, Bacillus massilinigeriensis, Bacillus phocaeensis and Bacillus tuaregi, five new species identified by culturomics. New Microbes New Infect..

[30] Hibbing M.E., Fuqua C., Parsek M.R., Peterson S.B. (2010). Bacterial competition: Surviving and thriving in the microbial jungle. Nat. Rev. Microbiol..

[31] Marcolefas E., Leung T., Okshevsky M., McKay G., Hignett E., Hamel J., Aguirre G., Blenner-Hasset O., Boyle B., Lévesque R.C. (2019). Culture-dependent bioprospecting of bacterial isolates from the Canadian high Arctic displaying antibacterial activity. Front. Microbiol..

[32] Hassell J.M., Ward M.J., Muloi D., Bettridge J.M., Robinson T.P., Kariuki S., Ogendo A., Kiiru J., Imboma T., Kang’ethe E.K. (2019). Clinically relevant antimicrobial resistance at the wildlife–livestock–human interface in Nairobi: An epidemiological study. Lancet Planet. Health.

