# Supplementary material for: Culturing Ancient Bacteria Carrying Resistance Genes from Permafrost and Comparative Genomics with Modern Isolates
Source: Microorganisms. 2020 Oct 3;8(10):1522. doi: 10.3390/microorganisms8101522 (PMC7600834; doi:10.3390/microorganisms8101522)
Supplement: Supplementary file 1 [file microorganisms-08-01522-s001.zip › Supplementary Data_and Tables.pdf]

**Supplementary Data**

# Culturing ancient bacteria carrying resistance genes from permafrost and comparative genomics with modern isolates

Pamela AFOUDA[1-2], Gregory DUBOURG[1-2], Anthony LEVASSEUR[1-2], Pierre-Edouard FOURNIER[2-3], Jeremy DELERCE[1], Oleg MEDIANNIKOV[1], Seydina M. DIENE [1-2], Jean-Pierre BAUDOIN[1], Daniel NAHON[4], Didier BOURLÈS[4], Jean-Marc ROLAIN[1-2] and Didier RAOULT[1-2 *]

[1] Aix Marseille Université, IRD, AP-HM, MEPHI, Marseille, France

[2] IHU Méditerranée Infection, Marseille, France

[3]UMR VITROME, SSA, Aix-Marseille Université, IRD, AP-HM, IHU-Méditerranée-Infection, Marseille France

[4] Aix-Marseille Univ., CNRS, IRD, INRAE, Coll France, UM 34 CEREGE, Technopôle de l'Environnement Arbois-Méditerranée, BP80, 13545 Aix-en-Provence, France

*__Corresponding author:__ Prof. Didier RAOULT

Adresse: Institut Hospitalo-Universitaire Méditerranée-Infection, 19-21 Boulevard Jean Moulin, 13385, Marseille cedex 05, France. Phone: +33 413 732 401 ; Fax: +33 413 732 402; Mail: didier.raoult@gmail.com

**ABBREVIATIONS**

ICP-OES : Inductively Coupled Plasma-Optical Emission Spectrometry

ASTER : Accélérateur pour les Sciences de la Terre, Environnement, Risques (Accelerator for Earth Sciences, Environment, Risks)

## SUPPLEMENTARY MATERIALS AND METHODS

### Specimen collection

Once extracted from the core drill, the sample was put immediately in a storage box, where from the entire piece about 3 kg was taken manually (manipulations were performed with a new linen gloves) and put in a sterile plastic bag. The sample was then transported frozen without being thawed. Examined macroscopically, this sample was composed to loam and pieces of dolomite and marl. The specimen was kept frozen (-20 °C) during the whole trip from Siberia to our laboratory in Marseille

### Culturomics

Seven solid media (supplementary Table 4) were incubated under 2 different atmospheres and at 3 different incubation temperatures. Besides, inoculation was performed both directly on the solid media and after pre-enrichment in blood culture bottle aerobic and anaerobic (supplementary Table 4). Follow-ups was performed every 3 days for 30 days. Culture media used included: 5% sheep blood-enriched columbia agar (COS) (bioMérieux, Marcy l'Etoile, France), modified-R-Medium [1], two halophilic media [2] (with pH=7 and pH=8.5) and three “minimal” media. The R-medium was consisted of a mixture of three solutions (A, B, C) [1]; it has been modified with sometimes a modification of the quantity of the elements contained in the solutions, the addition or subtraction of a component or a mutation of one solution component from one solution to another. Thus, solution A contained 15g casein hydrolysate proteose, 15g peptone, 10g yeast extract (Sigma-Aldrich), 2g α-ketoglutarate (Sigma-Aldrich), 5g sodium chloride (Merck Health), 10g glucose (MP Biomedicals, Illkirch, France), 0.5 g Na2S. The haemin (Sigma-Aldrich), α-ketoglutarate, sodium chloride and glucose were subtracted from solution B. The amount of distilled water was increased to 400 mL for solution C. 5% of sheep blood was added to the mixture of the three solutions after autoclave. The rest of the other elements remained identical as previously described [1]. The

minimal culture media was designed especially for this work in order to mimic environmental conditions. Three versions of this culture medium were performed in this study. The first version was composed of: 1g/L yeast extract, 1g/L peptone, 6 g/L NaCl, 1g/L K2HPO4, 15g/L agar, the pH was adjusted to 7; the second and third versions of minimal culture media have the same composition as the first, except for the amount of NaCl (1.5 g for second version) and pH (pH 8.5 for third version).

Cultures were performed under aerobic and anaerobic conditions with incubation at 3 different temperatures: room temperature (19°C±4), 30°C and 4°C, either in direct culture or after pre-enrichment in blood culture bottle containing 2ml sheep blood 2ml of rumen fluid previously filter sterilized through a 0.2 µm pore filter (Thermo Fisher Scientific, Villebon-sur-Yvette, France). The negative controls was also performed.

For each culture medium, we used each different type of incubation, culture and atmosphere. So, in total, it was used 84 different growing conditions, including 42 in aerobic and 42 in anaerobic conditions (Supplementary Table 4). All observed colonies were subcultured on columbia agar with 5% sheep blood (bioMérieux) for MALDI-TOF MS identification and before DNA extraction and 16S rRNA sequencing.

***Strain identification by MALDI-TOF MS and 16s rRNA sequencing***

MALDI-TOF MS analysis of different colonies was performed on a MicroFlex mass spectrometer (Bruker Daltonics, Leipzig, Germany) as previously described [3]. The acquired spectrum was then loaded into the MALDI Biotyper Software (Bruker Daltonics) and analyzed, as previously described [3] by using the standard pattern-matching algorithm, which compared the spectrum acquired with that present in the library (Bruker database and ours, constantly updated), including 7,463 species. Score values of ≥1.7 but <2 indicated identification beyond the genus level, and score values of ≥2.0 indicated identification at the species level. Scores of <1.7 were interpreted as not relevant.

Sequencing of 16S rRNA gene was performed for unidentified colonies. For DNA extraction, we used the EZ1 DNA Tissue Kit using Biorobot EZ1 Advanced XL (Qiagen, Courtaboeuf, France). The amplification of the 16s rRNA gene was carried out using PCR technology and universal primers FD1 and RP2 (Eurogentec, Angers, France). Afterwards, the purification, sequencing, and assembly of the amplified products were realized as previously described [4]. Sequences of 16S rRNA genes were confronted with those which are available in GenBank by BLASTn (http://www.ncbi.nlm.nih.gov/genbank/). When the percentage of identity was lower than 98.7%, the studied strain was considered as a new species [5].

## Antibiotic resistance

### *Phenotype of resistance*

The antibiograms were carried out according to the disks method to determine the sensitivity to antibiotics tested. As a prelude to the antibiotic susceptibility test, the strains were grown on Mueller-Hinton E (MH-E) agar for 24 to 48 hours. Then, from this fresh culture, an inoculum of 0.5 McFarland was prepared in saline (0.85% NaCl) and the antibiograms were performed on the same medium as recommended by EUCAST[6,7]. *Enterococcus faecalis* DSM 2570 and *Escherichia coli* DSM 1103 were used as quality controls. For Gram negative strains we tested 19 antibiotics representing 9 different families and for gram-positive we tested 16 antibiotics representing 11 different families (Table 5 B). For gram-negative strains the antibiotic disc panel used, included: Amoxicillin (25µg), Amoxicillin / Clavulanic Acid (30µg), Cefepime (30µg), Rifampicin (30µg), Cefalotin (30µg), Ceftriaxone (30µg), Piperacillin / Tazobactam (85µg), Imipenem (10 µg), Fosfomycin (50µg), Furane (300µg), Trimethoprim / Sulfamethoxazole (25µg), Amikacin (50µg), Ciprofloxacin (5µg), Doxycycline (30µg), Meropeneme (10µg), Gentamicine (500µg), Ticarcillin (75µg), Ticarcillin / clavulanic acid (75µg) and Colimycin (50µg). As for gram-positive strains, the antibiotic panel included: Penicillin G (10µg), Fosfomycin (30µg), Oxacillin (5µg),

Rifampicin (30µg), Clindamycin (15µg), Erythromycin (15µg), Pristinamycin (15µg), Gentamicin (15µg), Vancomycin (30µg), Teicoplanine (30µg), Doxycycline (30µg), Cefoxitine (30µg), Ciprofloxacin (5µg), Fusidic acid (10µg), Linezolid (30µg), Trimethoprim / Sulfamethoxazole (25µg) (Table 5 A and B).

**Permafrost dating**

***Mineralogical analysis***

*Interpretation:* Presence of dolomite. Therefore, the dating will be done through the determination of the in situ-produced cosmogenic nuclide $^{36}$Cl concentration accumulated within the carbonate minerals lattices as a result of nuclear reactions (spallation) between the primary and secondary cosmic-ray particles and, essentially, the calcium (Ca) and potassium (K ) constituting these minerals (insitu-production) and accumulating in the sample over time.

***Determination of the accumulated $^{36}$Cl concentration.***

*Protocol.*

- Sieving of the dried sample on a 50 µm sieve in order to eliminate the clay fraction;
- Decontamination of any atmospheric $^{36}$Cl (not produced in the carbonate minerals) component by MilliQ water (resistivity> 18.2 MOhms / cm) leaching followed by a partial dissolution (very partial in our case considering the quantity available) of the sample;
- Addition of the carrier (0.2919 g) whose characteristics are listed below

| **Spike 010813** | | |
|---|---|---|
| **Characteristics of the Spike 010813** | | Uncertainty |
| Measured average of the ratio $^{35}$Cl/$^{37}$Cl = | 918 | 4.4 |
| Measured average of the ratio $^{36}$Cl/$^{37}$Cl = | 2.717E-12 | 5.0E-13 |
| % $^{35}$ Cl = | 99.8912% | 0.0005% |
| % $^{37}$Cl = | 0.1088% | |
| Molar mass of Cl spike [g.mol$^{-1}$]= | 34.9710 | 0.0003 |
| Concentration of spike [mg/g] = | 6.92 | 0.05 |

- Total dissolution of dolomite and calcite (42.96 g) by addition of an excess of nitric acid;
- Filtration and removal of an aliquot to determine the concentration of Ca, K and Mg by ICP-OES (see below);
- Precipitation of Cl by addition of $AgNO_3$.
- Drying and measurements of the chlorine isotopic ratios on the French AMS national facility ASTER (see below).

*Determination of the concentration of target atoms by « Inductively Coupled Plasma-Optical Emission Spectrometry » (ICP-OES)*

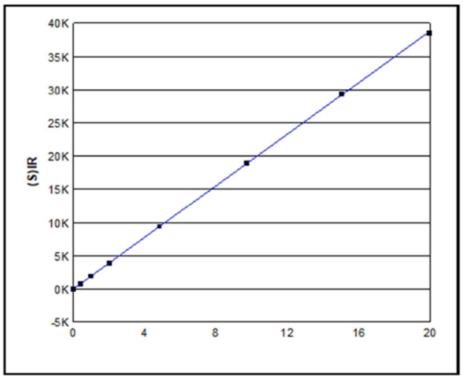


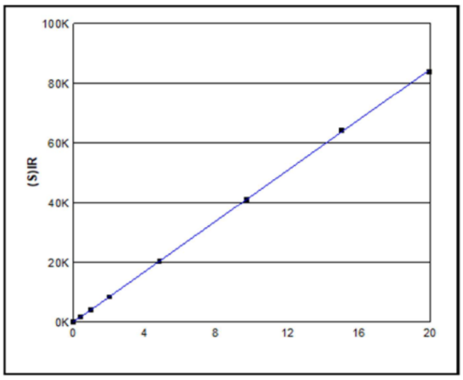


| | | | |
|---|---|---|---|
| **Element** | Ca | **Element Name:** | Ca |
| **Element** | Ca 315.887 nm | **Element** | Ca 317.933 nm |
| **Concentration** | ppm | **Concentration** | ppm |
| **Correlation:** | 0,99998 | Correlation: | 0,99999 |

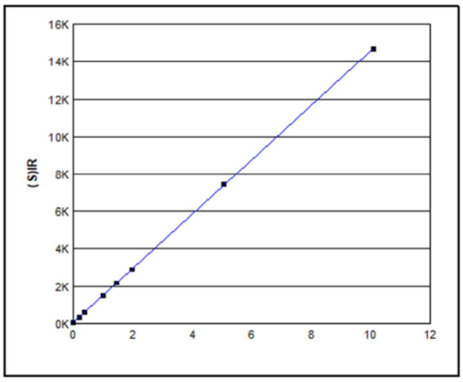


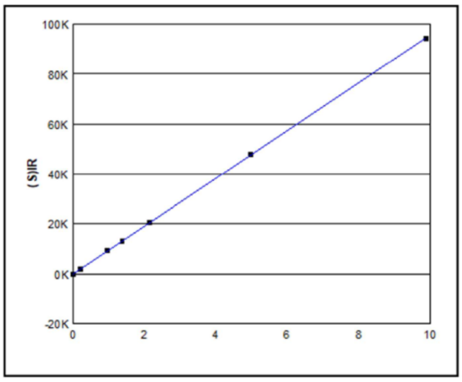


| | | | |
|---|---|---|---|
| **Element** | K | **Element Name:** | Mg |
| **Element** | K 766.490 nm | **Element** | Mg 285.213 nm |
| **Concentration** | ppm | **Concentration** | ppm |
| **Correlation:** | 0,99998 | **Correlation:** | 0,99998 |

**Sample Name** **Correction Factor**

Permafrost 1,00

**Concentration**

| **Element/Wavelength** | **Ca3158** | **Ca3179** | **K_7664** | **Mg2852** |
|---|---|---|---|---|
| **Units:** | ppm | ppm | ppm | ppm |
| **Avg. of Repeats:** | 5,884 | 5,869 | 0,0 | 2,996 |
| **Std Dev:** | 0,007 | 0,013 | 0,0 | 0,010 |
| **%RSD:** | 0,111 | 0,221 | 161,3 | 0,340 |
| **Repeat: 1** | 5,890 | 5,879 | 0,0 | 3,005 |
| **Repeat: 2** | 5,886 | 5,873 | 0,0 | 2,999 |
| **Repeat: 3** | 5,877 | 5,854 | 0,0 | 2,985 |

## *Determination of the in situ-produced $^{36}$Cl concentration.*

Measurements of the chlorine isotopic ratios ($^{36}Cl/^{35}Cl$, $^{36}Cl/^{37}Cl$, $^{35}Cl/^{37}Cl$) were

performed on the French AMS national facility ASTER, an accelerator mass spectrometer operating at 5 million volts (5 MV).

- ***Standards.***

| Standard name | Total Cl-36 counted events | | Measured Cl-36/Cl-35 | Measured Cl-36/Cl-37 | Measured Cl-35/Cl-37 |
|---|---|---|---|---|---|
| SM-CL-12 | 4504 | | 7.47378E-14 | 2.74945E-13 | 3.679 |
| SM-CL-12 | 4643 | | 7.26745E-14 | 2.67565E-13 | 3.682 |
| SM-CL-12 | 805 | | 7.42397E-14 | 2.75501E-13 | 3.711 |
| SM-CL-12 | 846 | | 6.81146E-14 | 2.50183E-13 | 3.673 |
| | | | | | |
| | | **Measured average ratio:** | 7.31838E-14 | 2.69411E-13 | 3.674268 |
| | | | | | |
| | | **Theoretical ratio:** | 1.428E-12 | 4.465E-12 | 3.127 |
| | | **Incertitude on the theoretical ratio (%):** | 1.5 | 1.5 | |
| | | | | | |
| | | **Correction factor:** | 19.51253 | 16.57321 | 0.8510539 |
| | | | | | |
| | | **Incertitude on the mean of the standard measurements (%):** | 0.9701 | 0.9698 | 0.2309 |

*In-house standard CEREGE : SM-CL-12*[8]
*External error: 2.74% ($^{36}Cl/^{35}Cl$), 2.13% ($^{36}Cl/^{37}Cl$) and 1.62% ($^{35}Cl/^{37}Cl$)*[9]

- ***Sample and chemical blank.***

| Sample name | Measured Cl-36/Cl-35 | Measured Cl-36/Cl-37 | Measured Cl-35/Cl-37 | Total Cl-36 counted events | Corrected Cl-36/Cl-35 | Uncertainty Cl-36/Cl-35 (%) | Corrected Cl-36/Cl-37 | Uncertainty Cl-36/Cl-37 (%) | Corrected Cl-35/Cl-37 | Uncertainty Cl-35/Cl-37 (%) |
|---|---|---|---|---|---|---|---|---|---|---|
| AgCl Blank | 1.12973E-16 | 4.17503E-16 | 3.696 | 6 | **2.20439E-15** | 30.1999 | **6.91936E-15** | 30.1516 | **3.145** | 1.6457 |
| Permafrost | 4.82906E-15 | 1.98739E-14 | 4.115 | 197 | **9.42271E-14** | 7.7123 | **3.29374E-13** | 7.5172 | **3.502** | 1.6853 |
| Chemical blank | 2.19525E-16 | 1.29829E-13 | 591.409 | 14 | **4.28348E-15** | 22.5986 | **2.15168E-12** | 22.5328 | **503.321** | 2.1716 |

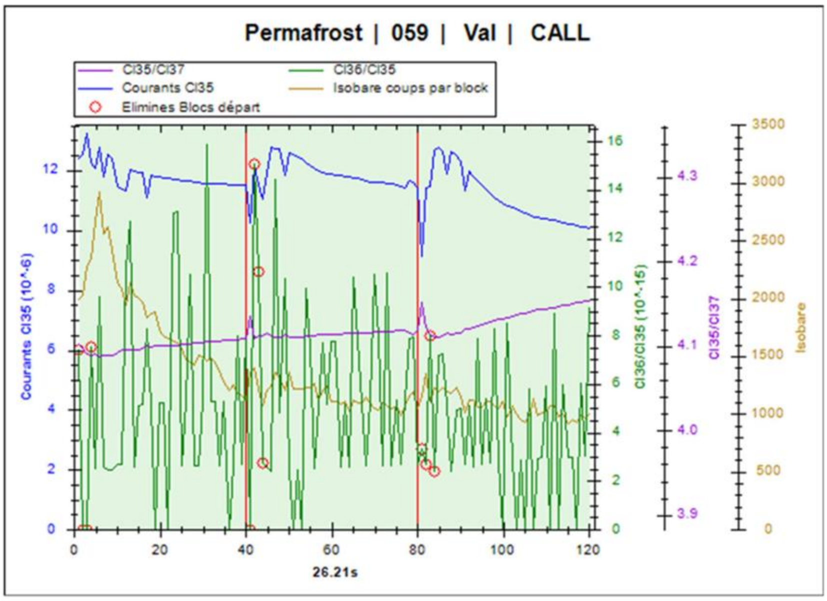


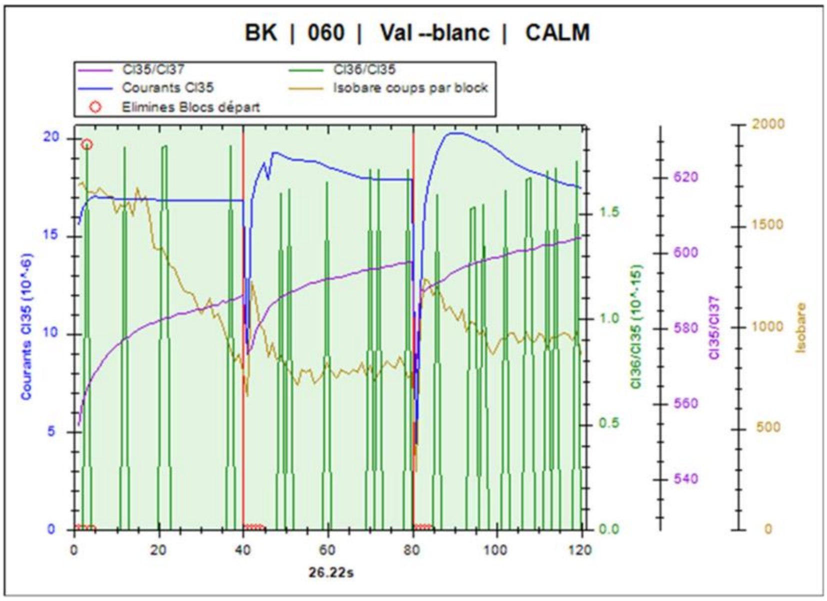


❖ ***$^{36}$Cl concentration.***

The in situ-produced $^{36}$Cl accumulated concentration calculated from all these measurements is: **(705 ± 114) x 10$^{3}$ at/g.**

***<u>Interpretation.</u>***

Such a high concentration of in situ-produced $^{36}$Cl accumulated at 10 meters depth implies a very long interaction time with particles from cosmic radiation. However, given the sampling depth (10 m), the determining parameter will be the density of the material overlying the

collected specimen. Indeed, this density must be such that the particles from cosmic radiation still have sufficient energy after passing through the overlying material to induce nuclear reactions producing $^{36}Cl$ from Ca (the concentration in K is negligible in the case studied, see above).

Assuming:

1- that the denudation rate affecting the area is negligible;

2- that the sample has always been at the depth from which it was taken;

3- that the cosmic radiation flux is constant over time;

the model allowing determining a cosmic ray exposure duration from the measured $^{36}Cl$ concentration converges to a solution for a density of the material covering over 10 m the sample of **1.18 g/cm³** leading to **a minimum exposure duration of (2.7 ± 0.4) million years**.

| Density : 1.18 g/cm³ | | | | |
|---|---|---|---|---|
| | $^{36}Cl$ concentration ($10^3$ atoms $^{36}Cl$/g) | $^{36}Cl$ uncertainty ($10^3$ atoms $^{36}Cl$/g) | Cosmic ray exposure duration at 10 m (million years) | Duration uncertainty (million years) |
| Permafrost sample | 705 | 114 | 2.7 | 0.4 |

Considering the characteristic density of different soil types (see table below), which depends both on (i) the abundance of the sandy fraction (reduced in our case) which induces a density increase),

and, (ii) the humidity rate (high in our case) which implies a density decrease), a density of 1.18 g/cm3 for the material covering the sample at 10 m depth seems entirely reasonable.

| | | | | | |
|---|---|---|---|---|---|
| Density (g/cm³) | 1.6 | 1.4 | 1.4 | 1.3 | 1.4 |

| Soil features | Sand, loamy sand, sandy loam | sandy loam, loam | Sand-clay loam, loam, clay loam | Limons, silt loam | silt loam, silty clay |
|---|---|---|---|---|---|

One can argue that the density used in this calculation is somehow low but due to the high water (ice) content in our sample (density of ice is ~0.92 g/cm3 between 0 and minus 20 degrees Celsius) and the muddy structure of the sample (bulk density of saturated muddy soil can vary from 1 to 1.3 g/$cm^3$ [10], a density of 1.18 g/cm3 for the material covering the sample at 10 m depth seems entirely reasonable. Considering a higher density will increase the sample shielding to cosmic rays and therefore will yield to an older age.

**Genomic study**

***DNA extraction***

gDNA of each strain was extracted in two steps :A mechanical treatment was first performed by glass beads acid washed (G4649-500g Sigma) using a FastPrep BIO 101 instrument (Qbiogene, Strasbourg, France) at maximum speed (6.5m/s) for 90s. Then after 2-2.5 hours lysozyme incubation at 37°C , DNA was extracted on the  EZ1 biorobot (Qiagen) with EZ1 DNA tissues kit. The elution volume is 50µL. gDNA was quantified by a Qubit assay with the high sensitivity kit (Life technologies, Carlsbad, CA, USA).  Quantified concentrations for the gDNAs of each strain are listed in supplementary Table 1.

***Genome sequencing***

**Genome sequencing  *Achromobacter insolitus* G1433b (Permafrost)**

Genomic DNA was sequenced on the MiSeq Technology (Illumina Inc, San Diego, CA, USA) with the 2 paired end. The paired end strategies were barcoded in order to be mixed respectively with 14 and 15 others genomic projects prepared with the Nextera XT DNA sample prep kit (Illumina).

To prepare the paired end library, dilution was performed to require 1ng of each genome as input. The « tagmentation » step fragmented and tagged the DNA. Then limited cycle PCR

amplification (12 cycles) completed the tag adapters and introduced dual-index barcodes. After purification on AMPure XP beads (Beckman Coulter Inc, Fullerton, CA, USA), the libraries were then normalized on specific beads according to the Nextera XT protocol (Illumina). Normalized libraries were pooled for sequencing on the MiSeq. Automated cluster generation and paired end sequencing with dual index reads were performed in a single 39-hours run in 2x250-bp.This library was loaded on two flowcells

Total information of 1.8 and 11.4 Gb was obtained from a 183 and 1348 k/mm2 cluster density with a cluster passing quality control filters of 91.4 and 87 %. Within this run, the index representation for *Achromobacter insolitus* was determined to 4.08 and 8.41%. The 142 184 and 1 861 354 paired end reads were filtered according to the read qualities.

The 2 paired end raw data were mixed to generate an assembly of the genome.

**Genome sequencing *Bacillus idriensis* G1436 (Permafrost): 1PE + 1 MinIon**

Genomic DNA was sequenced on the MiSeq Technology (Illumina Inc, San Diego, CA, USA) with the paired end strategy and was barcoded in order to be mixed respectively with 18 others genomic projects prepared with the Nextera XT DNA sample prep kit (Illumina) To prepare the paired end library, dilution was performed to require 1ng of each genome as input to prepare the paired end library. The « tagmentation » step fragmented and tagged the DNA. Then limited cycle PCR amplification (12 cycles) completed the tag adapters and introduced dual-index barcodes. After purification on AMPure XP beads (Beckman Coulter Inc, Fullerton, CA, USA), the libraries were then normalized on specific beads according to the Nextera XT protocol (Illumina). Normalized libraries were pooled into a single library for sequencing on the MiSeq. The pooled single strand library was loaded onto the reagent cartridge and then onto the instrument along with the flow cell. Automated cluster generation and paired end sequencing with dual index reads were performed in a single 39-hours run in 2x250-bp.

Total information of 8.6 Gb was obtained from a 963 k/mm2 cluster density with a cluster passing quality control filters of 89.9%. Within this run, the index representation for Bacillus idriensis was determined to 6.68%. The 1 105 836 paired end reads were filtered according to the read qualities.

Oxford Nanopore approach was performed on 1D genomic DNA sequencing for the MinIon device using SQK-LSK108 kit.Library was constructed from 1.5µg genomic DNA without fragmentation and end repair. Adapters were ligated to both ends of genomic DNA. After purification on AMPure XP beads (Beckman Coulter Inc, Fullerton, CA, USA), the library was quantified by a Qubit assay with the high sensitivity kit (Life technologies, Carlsbad, CA, USA). 92.52 ng adapted and tethered as library was loaded on the flow cell via the SpotON port.

1056 activ pores were detected for the sequencing and the workflow WIMP was choosen for bioinformatic analysis in live. After 35 minutes as run time and end life of the flowcell, 41 150 reads as raw data were generated.

**Genome sequencing *Bacillus idriensis* G1763 : 1PE**

Genomic DNA was sequenced on the MiSeq Technology (Illumina Inc, San Diego, CA, USA) with the paired end strategiy and was barcoded in order to be mixed respectively with 18 others genomic projects prepared with the Nextera XT DNA sample prep kit (Illumina) To prepare the paired end library, dilution was performed to require 1ng of each genome as input to prepare the paired end library. The « tagmentation » step fragmented and tagged the DNA. Then limited cycle PCR amplification (12 cycles) completed the tag adapters and introduced dual-index barcodes. After purification on AMPure XP beads (Beckman Coulter Inc, Fullerton, CA, USA), the libraries were then normalized on specific beads according to the Nextera XT protocol (Illumina). Normalized libraries were pooled into a single library for sequencing on the MiSeq. The pooled single strand library was loaded onto the reagent

cartridge and then onto the instrument along with the flow cell. Automated cluster generation and paired end sequencing with dual index reads were performed in a single 39-hours run in 2x250-bp.

Total information of  8.7 Gb was obtained from a 927K/mm2 cluster density with a cluster passing quality control filters of 94.1 %. Within this run, the index representation for Bacillus idriensis was determined to 4.74%. The 793 176 paired end reads were filtered according to the read qualities.

**Genome sequencing *Bacillus idriensis* G1764 : 1PE**

Genomic DNA was sequenced on the MiSeq Technology (Illumina Inc, San Diego, CA, USA) with the paired end  strategiy and was barcoded in order to be mixed respectively with 18 others genomic projects prepared with the Nextera XT DNA sample prep kit (Illumina) To prepare the paired end library, dilution was performed to require 1ng of each genome as input to prepare the paired end library. The « tagmentation » step fragmented and tagged the DNA. Then limited cycle PCR amplification (12 cycles) completed the tag adapters and introduced dual-index barcodes. After purification on AMPure XP beads (Beckman Coulter Inc, Fullerton, CA, USA), the libraries were then normalized on specific beads according to the Nextera XT protocol (Illumina). Normalized libraries were pooled into a single library for sequencing on the MiSeq. The pooled single strand library was loaded onto the reagent cartridge and then onto the instrument along with the flow cell. Automated cluster generation and paired end sequencing with dual index reads were performed in a single 39-hours run in 2x250-bp.

Total information of 8.7 Gb was obtained from a 927K/mm2 cluster density with a cluster passing quality control filters of 94.1 %. Within this run, the index representation for Bacillus idriensis was determined to 4.88%. The 816 977 paired end reads were filtered according to the read qualities.

**Genome sequencing *Brevundimonas aurantiaca* G1452 (Permafrost): 1MP+2PE+ 1 MinIon**

Genomic DNA was sequenced on the MiSeq Technology (Illumina Inc, San Diego, CA, USA) with the mate pair strategy. The gDNA was barcoded in order to be mixed with 11 others projects with the Nextera Mate Pair sample prep kit (Illumina).

The mate pair library was prepared with 1.5µg of genomic DNA using the Nextera mate pair Illumina guide. The genomic DNA sample was simultaneously fragmented and tagged with a mate pair junction adapter. The pattern of the fragmentation was validated on an Agilent 2100 BioAnalyzer (Agilent Technologies Inc, Santa Clara, CA, USA) with a DNA 7500 labchip. The DNA fragments ranged in size from 1.5 kb up to 11kb with an optimal size at 8.042 kb. No size selection was performed and 600 ng of tagmented fragments were circularized. The circularized DNA was mechanically sheared to small fragments with an optimal at 817 bp on the Covaris device S2 in T6 tubes (Covaris, Woburn, MA, USA). The library profile was visualized on a High Sensitivity Bioanalyzer LabChip (Agilent Technologies Inc, Santa Clara, CA, USA) and the final concentration library was measured at 9.03 nmol/l.

The libraries were normalized at 2nM and pooled. After a denaturation step and dilution at 15 pM, the pool of libraries was loaded onto the reagent cartridge and then onto the instrument along with the flow cell. Automated cluster generation and sequencing run were performed in a single 39-hours run in a 2x251-bp.

Total information of 8.2 Gb was obtained from a 932K/mm2 cluster density with a cluster passing quality control filters of 91 %. Within this run, the index representation for *Brevundimonas aurantiaca* was determined to 8.05%. The 1 305 582 paired end reads were filtered according to the read qualities.

Genomic DNA was sequenced on the MiSeq Technology (Illumina Inc, San Diego, CA, USA) with the 2 paired end. The paired end strategies were barcoded in order to be mixed

respectively with 14 and 15 others genomic projects prepared with the Nextera XT DNA sample prep kit (Illumina).

To prepare the paired end library, dilution was performed to require 1ng of each genome as input. The « tagmentation » step fragmented and tagged the DNA. Then limited cycle PCR amplification (12 cycles) completed the tag adapters and introduced dual-index barcodes. After purification on AMPure XP beads (Beckman Coulter Inc, Fullerton, CA, USA), the libraries were then normalized on specific beads according to the Nextera XT protocol (Illumina). Normalized libraries were pooled for sequencing on the MiSeq. Automated cluster generation and paired end sequencing with dual index reads were performed in a single 39-hours run in 2x250-bp.This library was loaded on two flowcells

Total information of 1.8 and 6.9 Gb was obtained from a 183 and 714 k/mm2 cluster density with a cluster passing quality control filters of 91.4 and 96.6 %. Within this run, the index representation for *Brevundimonas aurantiaca* was determined to 1.68 and 4.31%. The 58 613 and 576 036 paired end reads were filtered according to the read qualities

Oxford Nanopore approach was performed on 1D genomic DNA sequencing for the MinIon device using SQK-LSK108 kit.Library was constructed from 1.5µg genomic DNA without fragmentation and end repair. Adapters were ligated to both ends of genomic DNA. After purification on AMPure XP beads (Beckman Coulter Inc, Fullerton, CA, USA), the library was quantified by a Qubit assay with the high sensitivity kit (Life technologies, Carlsbad, CA, USA). 215.2ng adapted and tethered as library was loaded on the flow cell via the SpotON port.

1271 activ pores were detected for the sequencing and the workflow WIMP was choosen for bioinformatic analysis in live. After 1h31 as run time and end life of the flowcell, 39 720 reads as raw data were generated.

**Genome sequencing *Brevundimonas aurantiaca* G1603 : 1MP + 1MinIon**

Genomic DNA of was sequenced on the MiSeq Technology (Illumina Inc, San Diego, CA, USA) with the mate pair strategy. The gDNA was barcoded in order to be mixed with 11 others projects with the Nextera Mate Pair sample prep kit (Illumina).

The mate pair library was prepared with 1.5µg of genomic DNA using the Nextera mate pair Illumina guide. The genomic DNA sample was simultaneously fragmented and tagged with a mate pair junction adapter. The profile of the fragmentation was validated on an Agilent 2100 BioAnalyzer (Agilent Technologies Inc, Santa Clara, CA, USA) with a DNA 7500 labchip. The optimal size of obtained fragments was 7.07 kb. No size selection was performed and 600 ng of tagmented fragments were circularized. The circularized DNA was mechanically sheared to small fragments with an optimal at 367bp on the Covaris device S2 in T6 tubes (Covaris, Woburn, MA, USA). The library profile was visualized on a High Sensitivity Bioanalyzer LabChip (Agilent Technologies Inc, Santa Clara, CA, USA) and the final concentration library was measured at 6.7011 nmol/l. The libraries were normalized at 2 nM, pooled with 11 other projects, denatured and diluted at 25pM. Automated cluster generation and sequencing run were performed in a single 39-hours run in a 2x250-bp.

Total information of 2.9 Gb was obtained from a 290 K/mm2 cluster density with a cluster passing quality control filters of 98.5 %. Within this run, the index representation for *Brevundimonas aurantiaca* was determined to 7.43 %. The 428 609 paired end reads were filtered according to the read qualities.These reads were trimmed then assembled

Oxford Nanopore approach was performed on 1D genomic DNA sequencing for the MinIon device using SQK-LSK108 kit.Library was constructed from 1.5µg genomic DNA without fragmentation and end repair. Adapters were ligated to both ends of genomic DNA. After purification on AMPure XP beads (Beckman Coulter Inc, Fullerton, CA, USA), the library was quantified by a Qubit assay with the high sensitivity kit (Life technologies, Carlsbad, CA,

USA). 210.4 ng adapted and tethered as library was loaded on the flow cell via the SpotON port.

259 activ pores were detected for the sequencing and the workflow WIMP was choosen for bioinformatic analysis in live. After 5h33 as run time and end life of the flowcell, 38 292 reads as raw data were generated.

**<u>Genome sequencing *Brevundimonas aurantiaca* G1765 : 1PE</u>**

Genomic DNA was sequenced on the MiSeq Technology (Illumina Inc, San Diego, CA, USA) with the paired end strategiy and was barcoded in order to be mixed respectively with 18 others genomic projects prepared with the Nextera XT DNA sample prep kit (Illumina) To prepare the paired end library, dilution was performed to require 1ng of each genome as input to prepare the paired end library. The « tagmentation » step fragmented and tagged the DNA. Then limited cycle PCR amplification (12 cycles) completed the tag adapters and introduced dual-index barcodes. After purification on AMPure XP beads (Beckman Coulter Inc, Fullerton, CA, USA), the libraries were then normalized on specific beads according to the Nextera XT protocol (Illumina). Normalized libraries were pooled into a single library for sequencing on the MiSeq. The pooled single strand library was loaded onto the reagent cartridge and then onto the instrument along with the flow cell. Automated cluster generation and paired end sequencing with dual index reads were performed in a single 39-hours run in 2x250-bp.

Total information of 8.7 Gb was obtained from a 927K/mm2 cluster density with a cluster passing quality control filters of 94.1 %. Within this run, the index representation for *Brevundimonas aurantiaca* was determined to 4.97%. The 832 008 paired end reads were filtered according to the read qualities.

**<u>Genome sequencing *Brevundimonas aurantiaca* G1737: 1PE</u>**

Genomic DNA was sequenced on the MiSeq Technology (Illumina Inc, San Diego, CA, USA) with the paired end  strategiy and was barcoded in order to be mixed respectively with 24 others genomic projects prepared with the Nextera XT DNA sample prep kit (Illumina) To prepare the paired end library, dilution was performed to require 1ng of each genome as input to prepare the paired end library. The « tagmentation » step fragmented and tagged the DNA. Then limited cycle PCR amplification (12 cycles) completed the tag adapters and introduced dual-index barcodes. After purification on AMPure XP beads (Beckman Coulter Inc, Fullerton, CA, USA), the libraries were then normalized on specific beads according to the Nextera XT protocol (Illumina). Normalized libraries were pooled into a single library for sequencing on the MiSeq. The pooled single strand library was loaded onto the reagent cartridge and then onto the instrument along with the flow cell. Automated cluster generation and paired end sequencing with dual index reads were performed in a single 39-hours run in 2x250-bp.

Total information of  10.3Gb was obtained from a 1179K/mm2 cluster density with a cluster passing quality control filters of 89.7 %. Within this run, the index representation for *Brevundimonas aurantiaca* was determined to 5.45%. The 1 086 624 paired end reads were filtered according to the read qualities.

**Genome sequencing  *Janibacter melonis* G1437 (Pemafrost): 1PE + 1MinIon**

Genomic DNA was sequenced on the MiSeq Technology (Illumina Inc, San Diego, CA, USA) with the paired end  strategiy and was barcoded in order to be mixed respectively with 18 others genomic projects prepared with the Nextera XT DNA sample prep kit (Illumina) To prepare the paired end library, dilution was performed to require 1ng of each genome as input to prepare the paired end library. The « tagmentation » step fragmented and tagged the DNA. Then limited cycle PCR amplification (12 cycles) completed the tag adapters and introduced dual-index barcodes. After purification on AMPure XP beads (Beckman Coulter

Inc, Fullerton, CA, USA), the libraries were then normalized on specific beads according to the Nextera XT protocol (Illumina). Normalized libraries were pooled into a single library for sequencing on the MiSeq. The pooled single strand library was loaded onto the reagent cartridge and then onto the instrument along with the flow cell. Automated cluster generation and paired end sequencing with dual index reads were performed in a single 39-hours run in 2x250-bp.

Total information of 8.6 Gb was obtained from a 963 k/mm2 cluster density with a cluster passing quality control filters of  89.9%. Within this run, the index representation for Janibacter melonis was determined to 3.39%. The 560 645 paired end reads were filtered according to the read qualities.

Oxford Nanopore approach was performed on 1D genomic DNA sequencing for the MinIon device using SQK-LSK108 kit.Library was constructed from 1.5µg genomic DNA without fragmentation and end repair. Adapters were ligated to both ends of genomic DNA. After purification on AMPure XP beads (Beckman Coulter Inc, Fullerton, CA, USA), the library was quantified by a Qubit assay with the high sensitivity kit (Life technologies, Carlsbad, CA, USA). 264.8 ng adapted and tethered as library was loaded on the flow cell via the SpotON port.

780 activ pores were detected for the sequencing and the workflow WIMP was choosen for bioinformatic analysis in live. After 54 minutes as run time and end life of the flowcell, 43 923 reads as raw data were generated.

**Genome sequencing *Janibacter melonis* G1734 1PE + 1 MinIon**

Genomic DNA was sequenced on the MiSeq Technology (Illumina Inc, San Diego, CA, USA) with the paired end  strategiy and was barcoded in order to be mixed respectively with 24 others genomic projects prepared with the Nextera XT DNA sample prep kit (Illumina)

To prepare the paired end library, dilution was performed to require 1ng of each genome as input to prepare the paired end library. The « tagmentation » step fragmented and tagged the DNA. Then limited cycle PCR amplification (12 cycles) completed the tag adapters and introduced dual-index barcodes. After purification on AMPure XP beads (Beckman Coulter Inc, Fullerton, CA, USA), the libraries were then normalized on specific beads according to the Nextera XT protocol (Illumina). Normalized libraries were pooled into a single library for sequencing on the MiSeq. The pooled single strand library was loaded onto the reagent cartridge and then onto the instrument along with the flow cell. Automated cluster generation and paired end sequencing with dual index reads were performed in a single 39-hours run in 2x250-bp.

Total information of  10.3Gb was obtained from a 1179K/mm2 cluster density with a cluster passing quality control filters of 89.7 %. Within this run, the index representation for Janibacter melonis was determined to 1.54%. The 307 273 paired end reads were filtered according to the read qualities.

Oxford Nanopore approach was performed on 1D genomic DNA sequencing for the MinIon device using SQK-LSK108 kit.Library was constructed from 1.5µg genomic DNA without fragmentation and end repair. Adapters were ligated to both ends of genomic DNA. After purification on AMPure XP beads (Beckman Coulter Inc, Fullerton, CA, USA), the library was quantified by a Qubit assay with the high sensitivity kit (Life technologies, Carlsbad, CA, USA). 255.2 ng adapted and tethered as library was loaded on the flow cell via the SpotON port.

843 activ pores were detected for the sequencing and the workflow WIMP was choosen for bioinformatic analysis in live. After 1h40  as run time and end life of the flowcell, 41 260 reads as raw data were generated.

**Genome sequencing *Janibacter melonis* G1766: 1PE**

Genomic DNA was sequenced on the MiSeq Technology (Illumina Inc, San Diego, CA, USA) with the paired end strategiy and was barcoded in order to be mixed respectively with 18 others genomic projects prepared with the Nextera XT DNA sample prep kit (Illumina) To prepare the paired end library, dilution was performed to require 1ng of each genome as input to prepare the paired end library. The « tagmentation » step fragmented and tagged the DNA. Then limited cycle PCR amplification (12 cycles) completed the tag adapters and introduced dual-index barcodes. After purification on AMPure XP beads (Beckman Coulter Inc, Fullerton, CA, USA), the libraries were then normalized on specific beads according to the Nextera XT protocol (Illumina). Normalized libraries were pooled into a single library for sequencing on the MiSeq. The pooled single strand library was loaded onto the reagent cartridge and then onto the instrument along with the flow cell. Automated cluster generation and paired end sequencing with dual index reads were performed in a single 39-hours run in 2x250-bp.

Total information of 8.7 Gb was obtained from a 927K/mm2 cluster density with a cluster passing quality control filters of 94.1 %. Within this run, the index representation for Janibacter melonis was determined to 4.74%. The 793 176 paired end reads were filtered according to the read qualities

**Genome sequencing *Kocuria rhizophila* G1424 (Permafrost): 2PE**

Genomic DNA was sequenced on the MiSeq Technology (Illumina Inc, San Diego, CA, USA) with the 2 paired end. The paired end strategies were barcoded in order to be mixed respectively with 18 and 15 others genomic projects prepared with the Nextera XT DNA sample prep kit (Illumina).

To prepare the paired end library, dilution was performed to require 1ng of each genome as input. The « tagmentation » step fragmented and tagged the DNA. Then limited cycle PCR amplification (12 cycles) completed the tag adapters and introduced dual-index barcodes.

After purification on AMPure XP beads (Beckman Coulter Inc, Fullerton, CA, USA), the libraries were then normalized on specific beads according to the Nextera XT protocol (Illumina). Normalized libraries were pooled for sequencing on the MiSeq. Automated cluster generation and paired end sequencing with dual index reads were performed in a single 39-hours run in 2x250-bp.This library was loaded on two flowcells

Total information of 8.6 and 11.4 Gb was obtained from a 963 and 1348 k/mm2 cluster density with a cluster passing quality control filters of 89.9 and 87 %. Within this run, the index representation for Kocuria rhizophila was determined to 3.94 and 7.56%. The 651 836 and 1 671 896 paired end reads were filtered according to the read qualities.

The 2 paired end raw data were mixed to generate an assembly of the genome

**Genome sequencing *Microbacterium hydrocarbonoxydans* G1438 (Pemafrost): 1PE + 1MinIon**

Genomic DNA was sequenced on the MiSeq Technology (Illumina Inc, San Diego, CA, USA) with the paired end strategiy and was barcoded in order to be mixed respectively with 18 others genomic projects prepared with the Nextera XT DNA sample prep kit (Illumina)

To prepare the paired end library, dilution was performed to require 1ng of each genome as input to prepare the paired end library. The « tagmentation » step fragmented and tagged the DNA. Then limited cycle PCR amplification (12 cycles) completed the tag adapters and introduced dual-index barcodes. After purification on AMPure XP beads (Beckman Coulter Inc, Fullerton, CA, USA), the libraries were then normalized on specific beads according to the Nextera XT protocol (Illumina). Normalized libraries were pooled into a single library for sequencing on the MiSeq. The pooled single strand library was loaded onto the reagent cartridge and then onto the instrument along with the flow cell. Automated cluster generation and paired end sequencing with dual index reads were performed in a single 39-hours run in 2x250-bp.

Total information of 8.6 Gb was obtained from a 963 k/mm2 cluster density with a cluster passing quality control filters of  89.9%. Within this run, the index representation for Microbacterium hydrocarbonoxydans was determined to 3.87%. The 639 608 paired end reads were filtered according to the read qualities.

Oxford Nanopore approach was performed on 1D genomic DNA sequencing for the MinIon device using SQK-LSK108 kit.Library was constructed from 1.5µg genomic DNA without fragmentation and end repair. Adapters were ligated to both ends of genomic DNA. After purification on AMPure XP beads (Beckman Coulter Inc, Fullerton, CA, USA), the library was quantified by a Qubit assay with the high sensitivity kit (Life technologies, Carlsbad, CA, USA). 248.8 ng adapted and tethered as library was loaded on the flow cell via the SpotON port.

827 activ pores were detected for the sequencing and the workflow WIMP was choosen for bioinformatic analysis in live. After 1h47 as run time and end life of the flowcell, 43 923 reads as raw data were generated.

**Genome sequencing  *Paracoccus yeei* G1426 (Pemafrost): 2PE**

Genomic DNA was sequenced on the MiSeq Technology (Illumina Inc, San Diego, CA, USA) with the 2 paired end. The paired end strategies were barcoded in order to be mixed respectively with 18 and 15 others genomic projects prepared with the Nextera XT DNA sample prep kit (Illumina).

To prepare the paired end library, dilution was performed to require 1ng of each genome as input. The « tagmentation » step fragmented and tagged the DNA. Then limited cycle PCR amplification (12 cycles) completed the tag adapters and introduced dual-index barcodes. After purification on AMPure XP beads (Beckman Coulter Inc, Fullerton, CA, USA), the libraries were then normalized on specific beads according to the Nextera XT protocol (Illumina). Normalized libraries were pooled for sequencing on the MiSeq. Automated cluster

generation and paired end sequencing with dual index reads were performed in a single 39-hours run in 2x250-bp.This library was loaded on two flowcells

Total information of 8.6 and 11.4 Gb was obtained from a 963 and 1348 k/mm2 cluster density with a cluster passing quality control filters of 89.9 and 87 %. Within this run, the index representation for *Paracoccus yeei* was determined to 5.48 and 8.57%. The 906 329 and 1 896 724 paired end reads were filtered according to the read qualities.

The 2 paired end raw data were mixed to generate an assembly of the genome.

## SUPPLEMENTARY RESULTS AND DISCUSSION

### Physicochemical properties, dating and optimization of culture media

Physicochemical requirements were pH: 8.5; redox potential -87mV and salinity: 6g/L. The 7 culture media and the different conditions used (supplementary Table 4), were optimized for bacterial species culture in the permafrost sample. The age of permafrost determined from its in situ-produced cosmogenic nuclide $^{36}$Cl concentration has been estimated at ~2.7 million years.

### Microbial culturomics

The culture techniques were performed after cleaning and removing the outer layers of the carrots of the permafrost with sterile water to remove any contamination that might have occurred during handling before arrival in the laboratory. The 84 different conditions (supplementary Table 4) allowed us to test 7,015 bacterial colonies representing 28 bacterial species of which 20 were correctly identified by MALDI-TOF and 8 were identified by sequencing of rRNA 16S (Table 2). The bacteria obtained by 16S sequencing were: *Agrococcus baldri*, *Microbacterium hydrocarbonoxydans, Pedobacter quisquiliarum, Planomicrobium glaciei, Janibacter melonis, Pantoea massiliensis, Paenibacillus provencensis* and *Bacillus massiliglaciei*, this last being a new bacterial species

***Medium performance***

By comparing all cultures conditions, we found that the minimal medium (any atmosphere, pH and salinity combined) was the best conditions for bacterial isolation of this Siberian permafrost (Table 1).

In fact, of the 28 bacterial species, 22 (82.14 %) were obtained on minimal medium. The version of minimal medium for which NaCl concentration was 1.5 g/L and pH=7 allowed to isolate totality of these 22 bacterial species, whereas only 6/22 (27.27%) were recovered using the versions of minimal medium with different pH or NaCl concentrations (i.e., minimal medium NaCl 6, pH 7 and minimal medium NaCl 6, pH 8.5 respectively). Incubation at room temperature (i.e. 19°C±4) was more productive than that at 30°C (Table 1). Cultures performed at 4°C were all sterile and cultures on halophilic medium were also negative.

**Genomic analysis**

The genomes of permafrost species have been compared with three of their contemporaneous counterparts. For each species of permafrost, one of the closest species belonging to the same genus was also used to create an outgroup in the phylogenetic tree.

Based on the availability of at least three contemporary genomes and the elimination of a contamination hypothesis, seven bacterial species were selected for genomic studies. These species are: *Achromobacter insolitus, Bacillus idriensis, Brevundimonas aurantiaca, Janibacter melonis, Kocuria rhizophila, Microbacterium hydrocarbonoxydans* and *Paracoccus yeei.*

***Pan-genome***

***Core and accessory genes***

The numbers of total clusters of the pan-genomes were: 8,774; 8,156; 4,607; 4,648; 3,221; 6,825; 6,800 respectively for the species *Achromobacter insolitus, Bacillus idriensis, Brevundimonas aurantiaca, Janibacter melonis, Kocuria rhizophila, Microbacterium*

*hydrocarbonoxydans* and *Paracoccus yeei* .The core genome may represent about a quarter (1,449 genes/6,825=21.23% for *Microbacterium hydrocarbonoxydans*), a third (3421 genes/8,774=38.99% for *Achromobacter insolitus*; 2,670 genes/8,156=32.73% for *Bacillus idriensis* species) or about half (2,230 genes/4,607=48.40% for *Brevundimonas aurantiaca*; 2,584 genes/4,648=55.59% for *Janibacter melonis*; 1,686 genes/ 3,221=52.34% for *Kocuria rhizophila* and 3,171 genes/6,800=46.63% for *Paracoccus yeei*) of the total clusters (Supplementary Figure 3). The genomic content of permafrost species is clearly distinct from their contemporary counterparts.

***Unique genes***

For *Achromobacter insolitus*, the numbers of genes contained in the genomes are respectively of 5,929; 6,055; 6,032; 5,667 for non-permafrost strains: AB2, DSM23807, FDAARGOS88 and the permafrost strain. The number of genes that are unique to the genomes of these 4 strains are respectively of 119; 312; 663 and 2,143.

For *Bacillus idriensis*, the numbers of genes contained in the genomes are respectively of 4,759; 4,856; 4,722; 5,051 for non-permafrost strains: G715; G1763; G1764 and the permafrost strain. The number of genes that are unique to the genomes of these 4 strains are respectively of 597; 484; 341 and 1,005.

For *Brevundimonas aurantiaca*, the numbers of genes contained in the genomes are respectively of 3,275; 3,220; 3,127; 3,173 for non-permafrost strains: G1603, G1737, G1765 and the permafrost strain. The number of genes that are unique to the genomes of these 4 strains are respectively of 662; 377; 159 and 222.

For *Janibacter melonis*, the numbers of genes contained in the genomes are respectively 3,063; 3,873; 3,348; 3,261 for non-permafrost strains: CD114, G1734, G1766, and the permafrost strains. The number of genes that are unique to the genomes of these 4 strains are respectively of 261; 759; 178; 79.

For *Kocuria rhizophila* the numbers of genes contained in the genomes are respectively 2,306; 2,307; 2,461; 2,290 for non-permafrost strains: DC2201, FDAARGOS, UMB0131 and the permafrost strain. The number of genes that are unique to the genomes of these 4 strains are respectively of 2; 2; 342; 182.

For *Microbacterium hydrocarbonoxydans*, the numbers of genes contained in the genomes are respectively 3,419; 3,308; 3,653; 3,464 for non-permafrost strains: DSM16089, NBRC 103074, SA35 and the permafrost strain. The number of genes that are unique to the genomes of these 4 strains are respectively of 144; 49; 1,996; 1,249.

For *Paracoccus yeei*, the numbers of genes contained in the genomes are respectively 4,528; 4,624; 4,426; 4,500 for non-permafrost strains: CCUG32053, FDAARGOS, TT13 and permafrost strain. The number of genes that are unique to the genomes of these 4 strains are respectively of 727; 952; 315; 393.

It is therefore found that the proportions of unique genes are high for the permafrost strains compared to the contemporary strains for the species *Achromobacter insolitus* and *Bacillus idriensis*. For the other species, the highest proportions of unique genes are represented by one or more of the contemporary strains.

***Phylogeny and ancestral SNP***

Besides, the comparison of the evolutionary distances of bacterial strains of the same species with each other shows two cases:

(i) The case where the longest evolutionary distances are those shared between all contemporary strains and the permafrost strain. This is the case for the bacterial specie *Achromobacter insolitus* (Supplementary Figure 6A).

(i) The case where the longest evolutionary distances can also be shared on the one hand between the strain of permafrost and one or more of the contemporary strains and on the other hand between only the contemporary strains. This is observed for

bacterial species: *Bacillus idriensis* (Supplementary Figure 6B), *Brevundimonas aurantiaca* (Supplementary Figure 6C), *Janibacter melonis* (Supplementary Figure 6D), *Kocuria rhizophila* ( Supplementary Figure 6E), *Microbacterium hydrocarbonoxydans* (Supplementary Figure 6F) and *Paracoccus yeei* (Supplementary Figure 6G)

***Single-nucleotide polymorphism in the core genome in the same species' strains : AC versus CG***

Supplementary Table 3 shows the proportions of A and T mutated to G or C compared to those of G and C mutated to A or T in strains of the same species taken in pairs.

For the bacterial species *Bacillus idriensis* and *Microbacterium hydrocarbonoxydans,* the strain of permafrost compared to each contemporary strain reveals proportions of A and T mutated to G or C higher than those of G and C mutated to A or T. For these two bacterial species, the proportions of GC would therefore seem to be higher than those of the AT in the permafrost strain than in contemporary ones. The inverse scheme is observed for the strains of the species *Achromobacter insolitus* ie the proportions of G and C mutated to A or T are greater than those of A and T mutated to G or C (Supplementary Table 3).

For all strains of the others species, none of these two previous cases was found. The proportions of G and C mutated at A or T as well as A and T mutated to G or C can be superior to each other between the contemporary strains or between one or two contemporary strains and the permafrost strain (Supplementary Table 3).

**Supplementary Table 1. Bacterial strains used for genomic comparisons**

| | **Strains whose genomes are sequenced in the laboratory** | | | |
|---|---|---|---|---|
| **Species** | **Strain number/Project number** | **Strategy used** | **gDNA quantity** | **Isolation source** |
| *Achromobacter insolitus* | CSURP2857/G1433b | 2PE | 22 ng/µl | Permafrost |
| *Bacillus idriensis* | CSURP2855/G1436 | 1PE + 1 MinIon | 105 ng/µl | Permafrost |
| *Brevundimonas aurantiaca* | CSURP3513/G1452 | 1PE + 1 MinIon | 125 ng/µl | Permafrost |
| *Janibacter melonis* | CSURP2733/G1437 | 1PE + 1 MinIon | 61 ng/µl | Permafrost |
| *Kocuria rhizophila* | CSURP2672/G1424 | 2PE | 12 ng/µl | Permafrost |
| *Microbacterium hydrocarbonoxydans* | CSURP2596/G1438 | 1PE + 1 MinIon | 142 ng/µl | Permafrost |
| *Paracoccus yeei* | CSURP2668/G1426 | 2PE | 118 ng/µl | Permafrost |
| *Bacillus idriensis* | G715 | | | Human |
| *Bacillus idriensis* | CSURP7623/G1763 | 1PE | 199 ng/µl | Human |
| *Bacillus idriensis* | CSURP7624/G1764 | 1PE | 183 ng/µl | Human |
| *Brevundimonas aurantiaca* | CSURP666/G1603 | 1MP + 1MinIon | 107 ng/µl | Human |
| *Brevundimonas aurantiaca* | CSURP5651/G1737 | 1PE | 58 ng/µl | Human |
| *Brevundimonas aurantiaca* | CSURP7625/G1765 | 1PE | 93.2 ng/µl | Human |
| *Janibacter melonis* | CSURP690/G1734 | 1PE + 1 MinIon | 73.6 ng/µl | Human |
| *Janibacter melonis* | CSURP7627/ G1766 | 1PE | 108 ng/µl | Human |
| | **Species whose genomes are taken on NCBI genome** | | | |
| *Achromobacter insolitus* | strain AB2 | | | antarctic soil |
| *Achromobacter insolitus* | strain DSM23807 | | | Human |
| *Achromobacter insolitus* | strain LMG6003 | | | Human (leg wound) |
| *Janibacter melonis* | strain CD11-4 | | | Human (duodenal mucosa of celiac disease patient) |
| *Kocuria rhizophila* | strain DC2201 | | | soil |
| *Kocuria rhizophila* | strain FDAARGOS_302 | | | rhizosphere of narrowleaf cattail |
| *Kocuria rhizophila* | strain UMB0131 | | | Human (female urinary microbiome) |
| *Microbacterium hydrocarbonoxydans* | strain SA35 | | | rhizosphere |
| *Microbacterium hydrocarbonoxydans* | strain NBRC 103074 | | | oil-contaminated soil |
| *Microbacterium hydrocarbonoxydans* | strain DSM 16089 | | | Non Human |
| *Paracoccus yeei* | strain FDAARGOS-252 | | | Human (urine suprapubic aspirate) |
| *Paracoccus yeei* | strain TT13 | | | Human (human skin) |

| | | |
|---|---|---|
| *Paracoccus yeei* | strain CCUG 32053 | Human (eye) |
| | **Species close to our species permafrost ( taken on NCBI genome)** | |
| *Achromobacter aloeverae* | strain AVA-1 | Aloe vera |
| *Bacillus subtilis subsp. subtilis* | strain 168 | Human |
| *Brevundimonas subvibrioides* | strain ATCC 15264 | fresh water |
| *Janibacter indicus* | strain 0704P10-1 (= CGMCC 1.12511) | hydrothermal sediment |
| *Kocuria flava* | strain HO-9041 | air |
| *Microbacterium paraoxydans* | strain CF36 (=DSM15019) | Human (human blood) |
| *Paracoccus aminophilus* | strain DM-15 (=JCM 7686) | soil |

**PE: Paired-End; MP: Pate Pair**

**Supplementary Table 2.** Habitat of bacterial species isolated from permafrost

| **Permafrost strains** | **Known habitat** | **References** |
|---|---|---|
| *Achromobacter denitrificans* | - Human<br>- Environment (soil, water, mud, plants …) | [11–13]<br>[14] |
| *Achromobacter insolitus* | | |
| *Achromobacter pulmonis* | | [15] |
| *Achromobacter spanius* | | |
| *Acinetobacter baumannii* | - Human specimens,<br>- Natural environment | [16]<br>[17,18] |
| *Agrococcus baldri* | - Environment (air) | [19] |
| *Bacillus idriensis* | - Human<br>- Environment (Permafrost) | [16,20–22] |
| *Bacillus megaterium* | | |
| *Bacillus massilioglaciei* | | |
| *Bacillus simplex* | | |
| *Brevundimonas aurantiaca* | - Environment<br>- Permafrost-affected soils (*Brevundimonas* spp) | [23]<br>[22] |
| *Enterobacter cloacae* | - Human<br>- Environement (including Permafrost) | [24]<br>[16] |
| *Janibacter melonis* | - Cold environment : isolated from from Arctic sea ice, Canada Basin | [25] |
| *Kocuria rhizophila* | - Rhizoplane<br>- Environement (including Permafrost) | [26]<br>[21] |
| *Microbacterium hydrocarbonoxydans* | - Oil-contaminated soil<br>- Permafrost (*Microbacterium spp*) | [27]<br>[28] |
| *Micrococcus luteus* | - Human<br>- Environment (Amber, Permafrost) | [29]<br>[30]<br>[21] |
| *Paenibacillus provencensis* | - Human<br>- Environment (including Permafrost)Permafrost | [31]<br>[21,22] |
| *Paenibacillus urinalis* | | |
| *Pantoea massiliensis* | - Human | Lab data |
| *Pantoea septica* | - Human | [32] |
| *Paracoccus yeei* | - Human<br>- Arctic sea ice, Canada Basin (*Paracoccus spp*) | [33]<br>[25] |
| *Pedobacter quisquiliarum* | - Environment (sludge) | [34] |
| *Planomicrobium glaciei* | - Environment (Glacier) | [35] |
| *Sphingomonas paucimobilis* | - Human<br>- Environement (including Permafrost) | [36]<br>[20] |
| *Staphylococcus capitis* | - Human<br>- Environment<br>- Animal<br>- Food | [29,37] |
| *Staphylococcus epidermidis* | | |
| *Staphylococcus pasteuri* | | |
| *Staphylococcus saprophyticus* | | |

**Supplementary Table 3. Number of single-nucleotide polymorphisms in the core genome in the same species strains' : AC versus CG**

| *Achromobacter insolitus* | | | |
|---|---|---|---|
| **Strains** | GC->AT | AT->GC | Other |
| DSM23807 VS AB2 | 9669 | 9582 | 4375 |
| FDAARGOS VS AB2 | 8133 | 8290 | 3715 |
| FDAARGOS VS DSM23807 | 10069 | 10313 | 4799 |
| Permafrost VS DSM23807 | 159009 | 142699 | 188158 |
| Permafrost VS FDAARGOS | 159228 | 142674 | 188235 |
| Permafrost VS AB2 | 158994 | 142597 | 188037 |
| *Bacillus idriensis* | | | |
| **Strains** | GC->AT | AT->GC | Other |
| G1764 VS G1763 | 4402 | 4359 | 2258 |
| G715 VS G1763 | 133727 | 169363 | 92642 |
| G715 VS G1764 | 133802 | 169481 | 92735 |
| Permafrost VS G1764 | 133966 | 169761 | 92718 |
| Permafrost VS G715 | 9135 | 9251 | 5543 |
| Permafrost VS G1763 | 133917 | 169669 | 92689 |
| *Brevundimonas aurantiaca* | | | |
| **Strains** | GC->AT | AT->GC | Other |
| G1737 VS G1603 | 8434 | 7892 | 8011 |
| G1765 VS G1603 | 17164 | 16991 | 15823 |
| G1765 VS G1737 | 15397 | 15766 | 14707 |
| Permafrost VS G1737 | 15474 | 15823 | 14782 |
| Permafrost VS G1765 | 12231 | 12211 | 11268 |
| Permafrost VS G1603 | 17388 | 17195 | 16292 |
| *Janibacter melonis* | | | |
| **Strains** | GC->AT | AT->GC | Other |
| G1734 VS CD114 | 18982 | 16419 | 29319 |
| G1766 VS CD114 | 16474 | 15081 | 26753 |
| G1766 VS G1734 | 13592 | 14762 | 21387 |
| Permafrost VS G1734 | 15096 | 16171 | 25352 |
| Permafrost VS G1766 | 13346 | 13251 | 21627 |

| | | | |
|---|---|---|---|
| Permafrost VS CD114 | 16995 | 15507 | 28257 |
| ***Kocuria rhizophila*** | | | |
| **Strains** | GC->AT | AT->GC | Other |
| FDAARGOS VS DC2201 | 8 | 9 | 3 |
| UMB0131 VS DC2201 | 36186 | 43706 | 93609 |
| UMB0131 VS FDAARGOS | 36179 | 43698 | 93606 |
| Permafrost VS UMB0131 | 12268 | 12200 | 14380 |
| Permafrost VS DC2201 | 36294 | 43882 | 94245 |
| Permafrost VS FDAARGOS | 36287 | 43874 | 94242 |
| ***Microbacterium hydrocarbonoxydans*** | | | |
| **Strains** | GC->AT | AT->GC | Other |
| NBRC103074 VS DSM16089 | 0 | 0 | 1 |
| SA35 VS DSM16089 | 57576 | 59648 | 112189 |
| SA35 VS NBRC103074 | 57576 | 59648 | 112190 |
| Permafrost VS NBRC103074 | 45006 | 57103 | 94237 |
| Permafrost VS SA35 | 58702 | 68727 | 110859 |
| Permafrost VS DSM16089 | 45006 | 57103 | 94236 |
| ***Paracoccus yeei*** | | | |
| **Strains** | GC->AT | AT->GC | Other |
| FDAARGOS VS CCUG32053 | 17324 | 17909 | 11136 |
| TT13 VS CCUG32053 | 17246 | 17475 | 11329 |
| TT13 VS FDAARGOS | 16052 | 15696 | 10039 |
| Permafrost VS FDAARGOS | 16162 | 15799 | 10135 |
| Permafrost VS TT13 | 254 | 247 | 215 |
| Permafrost VS CCUG32053 | 17357 | 17579 | 11421 |

**Supplementary Table 4. Culture conditions of Siberian Permafrost sample. For each culture medium, each incubation temperature, each type of culture and atmosphere was used**

| Inoculation method | Media used | Incubation temperature | Atmosphere |
|---|---|---|---|
| Direct Culture<br><br>Preincubation blood culture bottle + blood+ rumen | Columbia agar<br><br>R-Medium<br><br>Halophile medium pH 7<br><br>Halophile medium pH 8.5<br><br>Minimal medium NaCl 1.5; pH 7<br><br>Minimal medium NaCl 6; pH 7<br><br>Minimal medium NaCl 6; pH 8.5 | Room temperature (19°C±4)<br><br>4°C<br><br>30°C | Aerobic<br><br>Anaerobic |

**Supplementary Table 5A. Antibiotic susceptibility testing results from gram-negative permafrost's strains and from their contemporary isolates available in the CSUR. R: resistant; S: susceptible; I: intermediate**. **Acquired resistances are highlighted in bold for permafrost strains.**

AMX: Amoxicillin ; AMC :Amoxicillin- clavulanate ; FEP :Cefepime ; CF :Cefalotin ; CRO :Ceftriaxone ; TZP : Piperacillin / Tazobactam ; IPM :Imipenem ; MEM :Meropenem ; TIC:Ticarcillin ; TCC :Ticarcillin- clavulanate ; AK : Amikacin ; GM :Gentamicin ; RA : Rifampicin ; FOS :Fosfomycin ; FT : Nitrofurantoin ; SXT:Trimethoprim/Sulfamethoxazole ; CIP: Ciprofloxacin ; DOX :Doxycycline ; CT : Colimycin

| **Strains / antibiotics** | AMX | AMC | FEP | CF | CRO | TZP | IPM | MEM | TIC | TCC | AK | GM | RA | FOS | FT | SXT | CIP | DOX | CT |
|---|---|---|---|---|---|---|---|---|---|---|---|---|---|---|---|---|---|---|---|
| *A. denitrificans permafrost* | R | R | R | R | R | S | S | S | S | S | S | S | R | R | **R** | **S** | **R** | S | S |
| *A. denitrificans CSUR P4838* | I | S | R | R | R | S | S | S | S | S | R | S | R | R | R | R | S | S | S |
| *A. denitrificans CSUR P9121* | R | S | R | R | R | S | S | S | S | S | S | S | R | R | R | S | S | S | S |
| *A. insolitus permafrost* | R | R | R | R | R | S | S | S | S | S | S | S | R | R | R | S | **R** | S | S |
| *A. insolitus CSUR P6430* | S | S | R | S | R | S | S | S | S | S | S | S | I | R | R | R | S | S | S |
| *A. insolitus CSUR P8677* | I | I | R | R | R | S | S | S | S | S | R | R | R | R | R | S | R | I | R |
| *A. pulmonis permafrost* | R | R | R | R | R | S | S | S | S | S | S | S | R | R | R | S | **R** | S | S |
| *A. spanius permafrost* | R | R | R | R | R | S | S | S | S | S | S | S | I | R | R | S | **R** | S | S |
| *A. spanius CSUR P539* | R | S | R | I | R | S | S | S | S | S | S | S | R | R | R | S | S | S | S |
| *A. baumannii permafrost* | R | R | **R** | R | R | **R** | I | S | R | R | R | S | R | R | R | R | **R** | S | S |
| *A. baumannii CSUR P1877* | R | R | R | R | R | R | R | R | R | R | R | S | I | R | R | R | R | R | S |
| *A. baumannii CSUR P1878* | R | R | R | R | R | R | R | R | R | R | R | S | I | R | R | R | R | R | S |
| *A. baumannii P1879* | R | R | R | R | R | R | R | R | R | R | R | S | I | R | R | R | R | R | S |
| *B. aurantiaca permafrost* | R | R | **R** | I | S | S | S | S | R | S | S | S | S | S | **R** | **R** | **R** | S | R |
| *E. cloacae permafrost* | R | R | S | R | S | S | S | S | S | S | S | S | R | S | **R** | S | S | S | S |
| *E. cloacae CSUR P1586* | R | R | S | R | S | S | S | S | S | S | S | S | R | S | R | S | S | S | S |
| *E. cloacae CSUR P1944* | R | R | R | R | R | R | I | S | R | R | S | S | R | R | R | R | R | R | S |
| *E. cloacae CSUR P2050* | R | R | R | R | R | R | R | R | R | R | S | S | R | S | R | R | R | S | S |
| *P. massiliensis permafrost* | R | R | S | I | S | S | S | S | S | S | S | S | R | **R** | **R** | S | S | S | S |
| *P. septica permafrost* | R | R | S | I | S | S | S | S | S | S | S | S | R | S | **R** | S | S | S | S |
| *P. septica CSUR P4907* | S | S | S | I | S | R | S | S | R | R | S | S | R | R | R | S | S | S | S |
| *P. septica CSUR P7571* | S | I | S | R | S | I | S | S | R | R | S | S | R | R | R | S | S | S | S |

| | | | | | | | | | | | | | | | | | | | |
|---|---|---|---|---|---|---|---|---|---|---|---|---|---|---|---|---|---|---|---|
| *P. septica CSUR P7887* | R | S | S | I | S | R | S | S | R | R | S | S | I | R | R | S | S | S | S |
| *P. yeei permafrost* | S | S | R | S | S | S | S | S | S | S | S | S | S | R | **R** | R | S | S | S |
| *P. yeei CSUR P8037* | S | S | R | S | S | I | S | S | S | S | S | S | S | R | S | R | S | S | S |
| *P. yeei CSUR P9414* | S | S | R | S | S | S | S | S | S | S | S | S | S | R | S | S | S | S | S |
| *P. yeei CSUR P9616* | S | S | R | S | S | I | S | S | S | S | S | S | S | R | S | R | S | S | S |
| *P. quisquiliarum permafrost* | R | S | S | R | R | S | S | S | S | S | S | S | S | R | S | S | S | S | R |
| *S. paucimobilis permafrost* | S | I | R | R | R | **R** | S | S | **R** | S | S | S | S | S | R | S | S | S | R |
| *S. paucimobilis CSUR P7590* | S | S | R | R | R | S | S | S | S | S | S | S | S | S | R | S | S | S | R |
| *S. paucimobilis CSUR P8007* | I | S | R | R | I | S | S | S | S | S | S | S | S | S | R | S | S | S | R |

**Supplementary Table 5B. Antibiotic susceptibility testing results from gram-positive permafrost's strains and from their contemporary isolates available in the CSUR. R: resistant; S: susceptible; I: intermediate**.

PG :Penicillin G ; OXA :Oxacillin ; FOX :Cefoxitin ; FOS: Fosfomycin ; RA :Rifampicin ; CLI :Clindamycin ; E : Erythromycin ; PT :Pristinamycin ; VA :Vancomycin ; TEC :Teicoplanin ; GM :Gentamycin : DOX :Doxycycline ; CIP :Ciprofloxacin ; FA :Fusic acid ; LZD : Linezolid ; SXT :Trimethoprim / Sulfamethoxazole

| **Strains / antibiotics** | PG | OXA | FOX | FOS | RA | CLI | E | PT | VA | TEC | GM | DOX | CIP | FA | LZD | SXT |
|---|---|---|---|---|---|---|---|---|---|---|---|---|---|---|---|---|
| *A. baldri permafrost* | S | S | S | S | S | S | S | S | S | S | S | S | S | S | S | S |
| *B. idriensis permafrost* | S | S | I | S | I | R | I | S | S | S | S | S | S | S | S | S |
| *B. idriensis CSUR P7624* | R | R | R | R | I | R | S | S | S | S | S | S | S | S | S | S |
| *B. idriensis CSUR P783* | R | R | R | S | I | R | S | S | S | S | S | S | S | S | S | S |
| *B. massiliglaciei permafrost* | S | S | S | R | S | S | S | S | S | S | S | S | S | R | S | S |
| *B. megaterium permafrost* | S | S | S | R | S | R | S | S | S | S | S | S | S | S | S | S |
| *B. megaterium CSUR P9393* | S | S | S | R | I | R | S | S | S | S | S | S | S | R | S | S |
| *B. megaterium CSUR P6976* | R | S | R | R | I | R | S | S | S | S | S | S | S | R | S | S |
| *B. megaterium CSUR P5156* | S | S | I | R | R | R | S | S | S | R | S | S | S | R | S | S |
| *B. simplex permafrost* | R | R | R | R | S | S | S | S | S | S | S | S | S | S | S | S |
| *B. simplex CSUR P9615* | R | R | R | S | I | S | R | S | S | S | S | S | S | R | S | S |
| *B. simplex CSUR P558* | R | R | R | S | I | S | R | S | S | S | S | S | S | R | S | S |
| *B. simplex CSUR P5196* | R | R | R | S | R | S | S | S | S | S | R | S | S | R | S | S |
| *J. melonis permafrost* | S | R | S | R | S | S | S | S | S | S | S | S | S | S | S | S |
| *K.rhizophila permafrost* | S | S | S | S | S | S | S | S | S | S | S | S | S | S | S | S |
| *K.rhizophila CSUR P8472* | S | S | S | S | S | S | S | S | S | S | R | S | S | S | S | S |
| *K.rhizophila CSUR P9116* | S | S | S | S | S | S | S | S | S | S | S | S | S | S | S | S |
| *K.rhizophila CSUR P8307* | S | R | S | R | S | S | I | S | S | S | S | S | S | S | S | S |

| | | | | | | | | | | | | | | | | |
|---|---|---|---|---|---|---|---|---|---|---|---|---|---|---|---|---|
| *M.hydrocarbonoxydans permafrost* | S | R | R | R | S | S | S | S | S | S | S | S | S | S | S | S |
| *M.luteus permafrost* | S | R | S | S | S | S | R | S | S | S | S | S | S | S | S | S |
| *M.luteus CSUR P8054* | S | R | S | S | S | S | S | S | S | S | S | S | S | S | S | S |
| *M.luteus CSUR P8591* | S | R | S | S | S | S | S | S | S | S | S | S | S | S | S | S |
| *M.luteus CSUR P9060* | S | R | S | S | S | S | S | S | S | S | S | S | S | S | S | S |
| *P.provencensis permafrost* | R | R | R | R | I | R | R | S | S | S | S | S | S | S | S | S |
| *P.provencensis CSUR P281* | S | R | R | R | S | R | S | S | S | S | S | S | S | S | S | S |
| *P.provencensis CSUR 793* | R | R | R | R | S | R | R | S | S | S | S | S | S | R | S | S |
| *P.urinalis permafrost* | R | R | R | R | I | R | R | I | S | S | S | S | S | R | S | S |
| *P.urinalis CSUR P6030* | R | R | R | R | I | R | R | S | S | S | S | S | S | S | S | S |
| *P.urinalis CSUR P280* | R | R | R | R | S | R | R | S | S | S | S | S | S | R | S | S |
| *P.glaciei permafrost* | S | S | R | S | S | S | S | S | S | S | S | S | S | S | S | S |
| *S. capitis permafrost* | R | S | I | R | S | S | S | S | S | S | S | S | S | S | S | S |
| *S. capitis CSUR P5933* | S | S | S | R | S | S | S | S | S | S | S | S | S | S | S | S |
| *S. capitis CSUR P6520* | R | R | R | R | S | S | S | S | S | S | R | S | S | S | S | S |
| *S. capitis CSUR P311* | R | R | R | S | R | S | S | S | S | S | R | S | S | R | S | S |
| *S. epidermidis permafrost* | R | S | R | R | I | S | S | S | S | S | S | S | S | S | S | R |
| *S. epidermidis CSUR P2761* | S | S | S | S | S | S | R | S | S | S | S | S | S | R | S | S |
| *S. epidermidis CSUR P2286* | R | S | S | S | S | S | R | S | S | S | S | I | S | R | S | S |
| *S. pasteuri permafrost* | R | S | S | R | I | S | S | S | S | S | S | S | S | S | S | S |
| *S. pasteuri CSUR P7522* | R | S | S | S | S | S | S | R | S | S | S | S | S | S | S | S |
| *S. pasteuri CSUR P8187* | S | S | S | S | S | S | S | S | S | S | S | S | S | S | S | S |
| *S. pasteuri CSUR P7813* | R | S | S | R | S | S | R | S | S | S | S | S | S | S | S | S |
| *S. saprophyticus permafrost* | S | S | R | R | S | S | R | S | S | S | S | I | S | R | S | S |
| *S. saprophyticus CSUR P7584* | S | S | S | R | S | S | R | S | S | S | S | S | S | R | S | S |

| | | | | | | | | | | | | | | | | |
|---|---|---|---|---|---|---|---|---|---|---|---|---|---|---|---|---|
| *S. saprophyticus CSUR P7602* | S | S | R | S | S | S | S | I | S | S | S | S | S | R | S | S |
| *S. saprophyticus CSUR P7607* | S | S | S | S | S | S | S | I | S | S | S | S | S | R | S | S |

References


1. Dione, N.; Khelaifia, S.; La Scola, B.; Lagier, J.C.; Raoult, D. A quasi-universal medium to break the aerobic/anaerobic bacterial culture dichotomy in clinical microbiology. *Clin. Microbiol. Infect. Off. Publ. Eur. Soc. Clin. Microbiol. Infect. Dis.* **2016**, *22*, 53–58, doi:10.1016/j.cmi.2015.10.032.
2. Seck, E.H.; Sankar, S.A.; Khelaifia, S.; Croce, O.; Robert, C.; Couderc, C.; Di Pinto, F.; Sokhna, C.; Fournier, P.-E.; Raoult, D.; et al. Noncontiguous finished genome sequence and description of *Planococcus massiliensis* sp. nov., a moderately halophilic bacterium isolated from the human gut. *New Microbes New Infect.* **2016**, *10*, 36–46, doi:10.1016/j.nmni.2015.12.006.
3. Seng, P.; Drancourt, M.; Gouriet, F.; La Scola, B.; Fournier, P.-E.; Rolain, J.M.; Raoult, D. Ongoing Revolution in Bacteriology: Routine Identification of Bacteria by Matrix-Assisted Laser Desorption Ionization Time-of-Flight Mass Spectrometry. *Clin. Infect. Dis.* **2009**, *49*, 543–551, doi:10.1086/600885.
4. Morel, A.-S.; Dubourg, G.; Prudent, E.; Edouard, S.; Gouriet, F.; Casalta, J.-P.; Fenollar, F.; Fournier, P.E.; Drancourt, M.; Raoult, D. Complementarity between targeted real-time specific PCR and conventional broad-range 16S rDNA PCR in the syndrome-driven diagnosis of infectious diseases. *Eur. J. Clin. Microbiol. Infect. Dis. Off. Publ. Eur. Soc. Clin. Microbiol.* **2015**, *34*, 561–570, doi:10.1007/s10096-014-2263-z.
5. Stackebrandt, E.; Ebers, J. Taxonomic parameters revisited: Tarnished gold standards. *Microbiol Today* **2006**, *8*, 6–9.
6. Citron, D.M.; Ostovari, M.I.; Karlsson, A.; Goldstein, E.J. Evaluation of the E test for susceptibility testing of anaerobic bacteria. *J. Clin. Microbiol.* **1991**, *29*, 2197–2203.
7. Matuschek, E.; Brown, D.F.J.; Kahlmeter, G. Development of the EUCAST disk diffusion antimicrobial susceptibility testing method and its implementation in routine microbiology laboratories. *Clin. Microbiol. Infect. Off. Publ. Eur. Soc. Clin. Microbiol. Infect. Dis.* **2014**, *20*, O255-266, doi:10.1111/1469-0691.12373.
8. Merchel, S.; Bremser, W.; Alfimov, V.; Arnold, M.; Aumaître, G.; Benedetti, L.; Bourlès, D.L.; Caffee, M.; Fifield, L.K.; Finkel, R.C.; et al. Ultra-trace analysis of 36Cl by accelerator mass spectrometry: an interlaboratory study. *Anal. Bioanal. Chem.* **2011**, *400*, 3125–3132, doi:10.1007/s00216-011-4979-2.
9. Braucher, R.; Keddadouche, K.; Aumaître, G.; Bourlès, D.L.; Arnold, M.; Pivot, S.; Baroni, M.; Scharf, A.; Rugel, G.; Bard, E. Chlorine measurements at the 5MV French AMS national facility ASTER: Associated external uncertainties and comparability with the 6MV DREAMS facility. *Nucl. Instrum. Methods Phys. Res. Sect. B Beam Interact. Mater. At.* **2018**, *420*, 40–45, doi:10.1016/j.nimb.2018.01.025.
10. Ladanyi, B. *An Introduction to Frozen Ground Engineering*; New York: Chapman & Hall, 1994; ISBN 0-412-98201-3.
11. Coenye, T.; Vancanneyt, M.; Falsen, E.; Swings, J.; Vandamme, P. Achromobacter insolitus sp. nov. and Achromobacter spanius sp. nov., from human clinical samples. *Int. J. Syst. Evol. Microbiol.* **2003**, *53*, 1819–1824, doi:10.1099/ijs.0.02698-0.
12. Vandamme, P.; Moore, E.R.B.; Cnockaert, M.; De Brandt, E.; Svensson-Stadler, L.; Houf, K.; Spilker, T.; Lipuma, J.J. Achromobacter animicus sp. nov., Achromobacter mucicolens sp. nov., *Achromobacter pulmonis* sp. nov. and *Achromobacter spiritinus* sp. nov., from human clinical samples. *Syst. Appl. Microbiol.* **2013**, *36*, 1–10, doi:10.1016/j.syapm.2012.10.003.
13. Spear, J.B.; Fuhrer, J.; Kirby, B.D. Achromobacter xylosoxidans (Alcaligenes xylosoxidans subsp. xylosoxidans) bacteremia associated with a well-water source: case report and review of the literature. *J. Clin. Microbiol.* **1988**, *26*, 598–599.

14. Amoureux, L.; Bador, J.; Fardeheb, S.; Mabille, C.; Couchot, C.; Massip, C.; Salignon, A.-L.; Berlie, G.; Varin, V.; Neuwirth, C. Detection of Achromobacter xylosoxidans in hospital, domestic, and outdoor environmental samples and comparison with human clinical isolates. *Appl. Environ. Microbiol.* **2013**, *79*, 7142–7149, doi:10.1128/AEM.02293-13.
15. Yang, S.; Wen, X.; Jin, H.; Wu, Q. Pyrosequencing Investigation into the Bacterial Community in Permafrost Soils along the China-Russia Crude Oil Pipeline (CRCOP). *PLOS ONE* **2012**, *7*, e52730, doi:10.1371/journal.pone.0052730.
16. Singh, P.; Singh, S.M.; Singh, R.N.; Naik, S.; Roy, U.; Srivastava, A.; Bölter, M. Bacterial communities in ancient permafrost profiles of Svalbard, Arctic. *J. Basic Microbiol.* **2017**, *57*, 1018–1036, doi:10.1002/jobm.201700061.
17. Antunes, L.C.S.; Visca, P.; Towner, K.J. *Acinetobacter baumanni*i: evolution of a global pathogen. *Pathog. Dis.* **2014**, *71*, 292–301, doi:10.1111/2049-632X.12125.
18. Bouvet, P.J.M.; Grimont, P.A.D. Taxonomy of the Genus *Acinetobacter* with the Recognition of *Acinetobacter baumannii* sp. nov., *Acinetobacter haemolyticus* sp. nov., *Acinetobacter johnsonii* sp. nov., and *Acinetobacter junii* sp. nov. and Emended Descriptions of Acinetobacter calcoaceticus and Acinetobacter lwoffii. *Int. J. Syst. Evol. Microbiol.* **1986**, *36*, 228–240, doi:10.1099/00207713-36-2-228.
19. Zlamala, C.; Schumann, P.; Kämpfer, P.; Rosselló-Mora, R.; Lubitz, W.; Busse, H.-J. *Agrococcus baldri* sp. nov., isolated from the air in the “Virgilkapelle” in Vienna. *Int. J. Syst. Evol. Microbiol.* **2002**, *52*, 1211–1216, doi:10.1099/00207713-52-4-1211.
20. Vishnivetskaya, T.A.; Petrova, M.A.; Urbance, J.; Ponder, M.; Moyer, C.L.; Gilichinsky, D.A.; Tiedje, J.M. Bacterial community in ancient Siberian permafrost as characterized by culture and culture-independent methods. *Astrobiology* **2006**, *6*, 400–414, doi:10.1089/ast.2006.6.400.
21. Steven, B.; Briggs, G.; McKay, C.P.; Pollard, W.H.; Greer, C.W.; Whyte, L.G. Characterization of the microbial diversity in a permafrost sample from the Canadian high Arctic using culture-dependent and culture-independent methods. *FEMS Microbiol. Ecol.* **2007**, *59*, 513–523, doi:10.1111/j.1574-6941.2006.00247.x.
22. Ganzert, L.; Bajerski, F.; Wagner, D. Bacterial community composition and diversity of five different permafrost-affected soils of Northeast Greenland. *FEMS Microbiol. Ecol.* **2014**, *89*, 426–441, doi:10.1111/1574-6941.12352.
23. Phylogeny and polyphasic taxonomy of *Caulobacter* species. Proposal o*f Maricaulis* gen. nov. with *Maricaulis maris* (Poindexter) comb. nov. as the type species, and emended description of the genera *Brevundimonas* and *Caulobacter* | Microbiology Society Available online: https://www.microbiologyresearch.org/content/journal/ijsem/10.1099/00207713-49-3-1053 (accessed on Sep 16, 2019).
24. Enterobacter | Description, Characteristics, Species, & Drug Resistance | Britannica.com Available online: https://www.britannica.com/science/Enterobacter (accessed on Sep 16, 2019).
25. Yu, Y.; Li, H.; Zeng, Y.; Chen, B. Extracellular enzymes of cold-adapted bacteria from Arctic sea ice, Canada Basin. *Polar Biol.* **2009**, *32*, 1539–1547, doi:10.1007/s00300-009-0654-x.
26. Kovács, G.; Burghardt, J.; Pradella, S.; Schumann, P.; Stackebrandt, E.; Màrialigeti, K. *Kocuria palustris* sp. nov. and *Kocuria rhizophila* sp. nov., isolated from the rhizoplane of the narrow-leaved cattail (Typha angustifolia). *Int. J. Syst. Evol. Microbiol.* **1999**, *49*, 167–173, doi:10.1099/00207713-49-1-167.
27. Schippers, A.; Bosecker, K.; Spröer, C.; Schumann, P. *Microbacterium oleivorans* sp. nov. and *Microbacterium hydrocarbonoxydans* sp. nov., novel crude-oil-degrading

Gram-positive bacteria. *Int. J. Syst. Evol. Microbiol.* **2005**, *55*, 655–660, doi:10.1099/ijs.0.63305-0.

28. Cheptsov, V.; Vorobyova, E.; Belov, A.; Pavlov, A.; Tsurkov, D.; Lomasov, V.; Bulat, S. Survivability of Soil and Permafrost Microbial Communities after Irradiation with Accelerated Electrons under Simulated Martian and Open Space Conditions. *Geosciences* **2018**, *8*, 298, doi:10.3390/geosciences8080298.
29. Chiller, K.; Selkin, B.A.; Murakawa, G.J. Skin Microflora and Bacterial Infections of the Skin. *J. Investig. Dermatol. Symp. Proc.* **2001**, *6*, 170–174, doi:10.1046/j.0022-202x.2001.00043.x.
30. Greenblatt, C.L.; Baum, J.; Klein, B.Y.; Nachshon, S.; Koltunov, V.; Cano, R.J. Micrococcus luteus -- survival in amber. *Microb. Ecol.* **2004**, *48*, 120–127, doi:10.1007/s00248-003-2016-5.
31. Roux, V.; Fenner, L.; Raoult, D. *Paenibacillus provencensis* sp. nov., isolated from human cerebrospinal fluid, and Paenibacillus urinalis sp. nov., isolated from human urine. *Int. J. Syst. Evol. Microbiol.* **2008**, *58*, 682–687, doi:10.1099/ijs.0.65228-0.
32. Brady, C.L.; Cleenwerck, I.; Venter, S.N.; Engelbeen, K.; De Vos, P.; Coutinho, T.A. Emended description of the genus Pantoea, description of four species from human clinical samples, *Pantoea septica* sp. nov., *Pantoea eucrina* sp. nov., *Pantoea brenneri* sp. nov. and *Pantoea conspicua* sp. nov., and transfer of *Pectobacterium cypripedii* (Hori 1911) Brenner et al. 1973 emend. Hauben et al. 1998 to the genus as P*antoea cypripedii* comb. nov. *Int. J. Syst. Evol. Microbiol.* **2010**, *60*, 2430–2440, doi:10.1099/ijs.0.017301-0.
33. Arias, M.A.; Clark, J. Paracoccus yeei as a cause of peritoneal dialysis peritonitis in the United Kingdom. *IDCases* **2019**, *15*, doi:10.1016/j.idcr.2019.e00486.
34. Zhang, B.; Liu, Z.-Q.; Zheng, Y.-G. Pedobacter quisquiliarum sp. nov., isolated from activated sludge. *Int. J. Syst. Evol. Microbiol.* **2018**, *68*, 438–442, doi:10.1099/ijsem.0.002531.
35. Zhang, D.-C.; Liu, H.-C.; Xin, Y.-H.; Yu, Y.; Zhou, P.-J.; Zhou, Y.-G. *Planomicrobium glaciei* sp. nov., a psychrotolerant bacterium isolated from a glacier. *Int. J. Syst. Evol. Microbiol.* **2009**, *59*, 1387–1390, doi:10.1099/ijs.0.002592-0.
36. Ryan, M.P.; Adley, C.C. *Sphingomonas paucimobilis*: a persistent Gram-negative nosocomial infectious organism. *J. Hosp. Infect.* **2010**, *75*, 153–157, doi:10.1016/j.jhin.2010.03.007.
37. Chesneau, O.; Morvan, A.; Grimont, F.; Labischinski, H.; el Solh, N. *Staphylococcus pasteuri* sp. nov., isolated from human, animal, and food specimens. *Int. J. Syst. Bacteriol.* **1993**, *43*, 237–244, doi:10.1099/00207713-43-2-237.
