# Supplementary material for: Culturing Ancient Bacteria Carrying Resistance Genes from Permafrost and Comparative Genomics with Modern Isolates
Source: Microorganisms. 2020 Oct 3;8(10):1522. doi: 10.3390/microorganisms8101522 (PMC7600834; doi:10.3390/microorganisms8101522)
Supplement: Supplementary file 1 [file microorganisms-08-01522-s001.zip › Supplementary Figures.pdf]

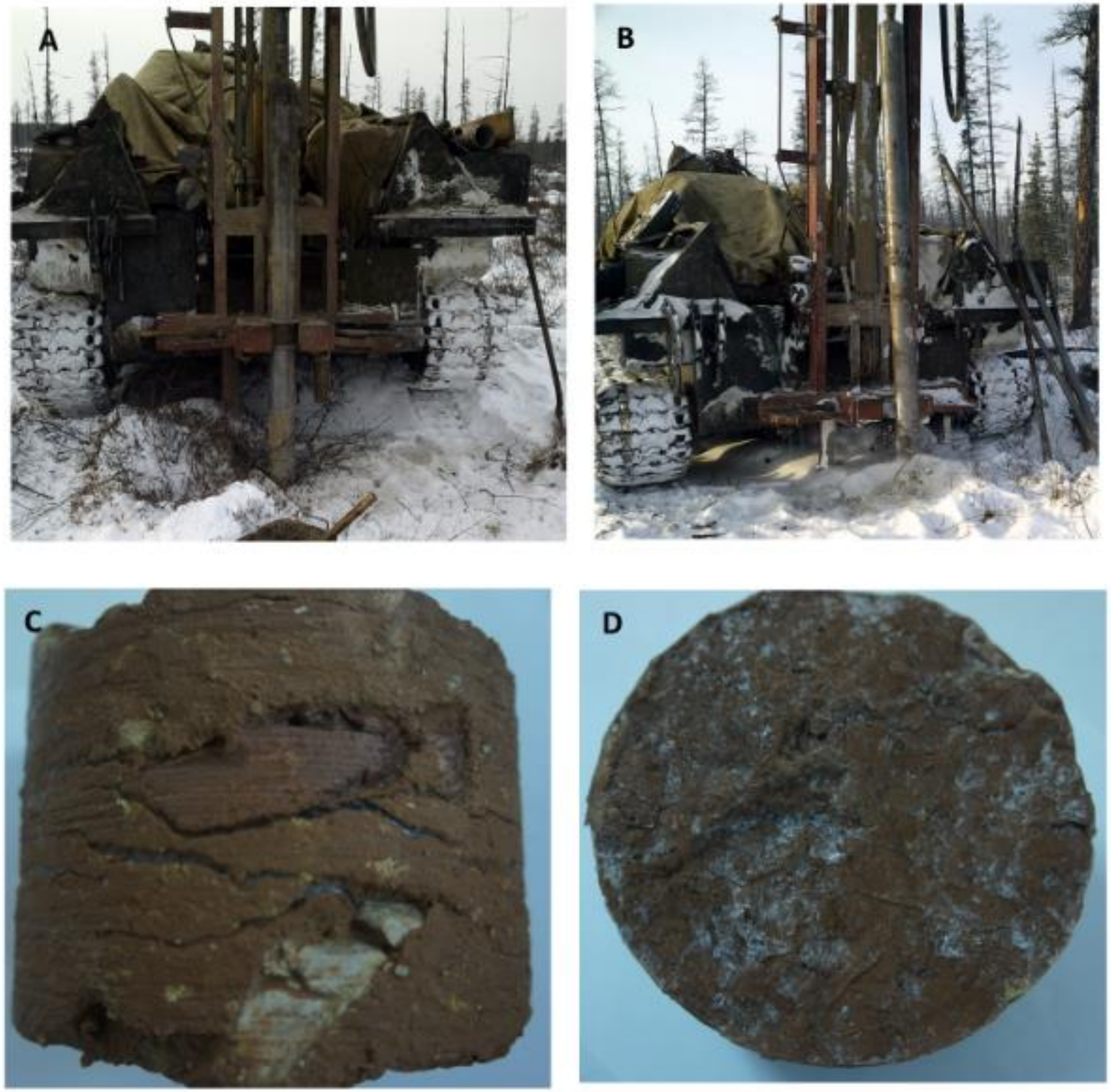

Supplementary Figure 1. Illustration of the permafrost collection process. A and B : core drill. C and D: permafrot specimen following shipment.

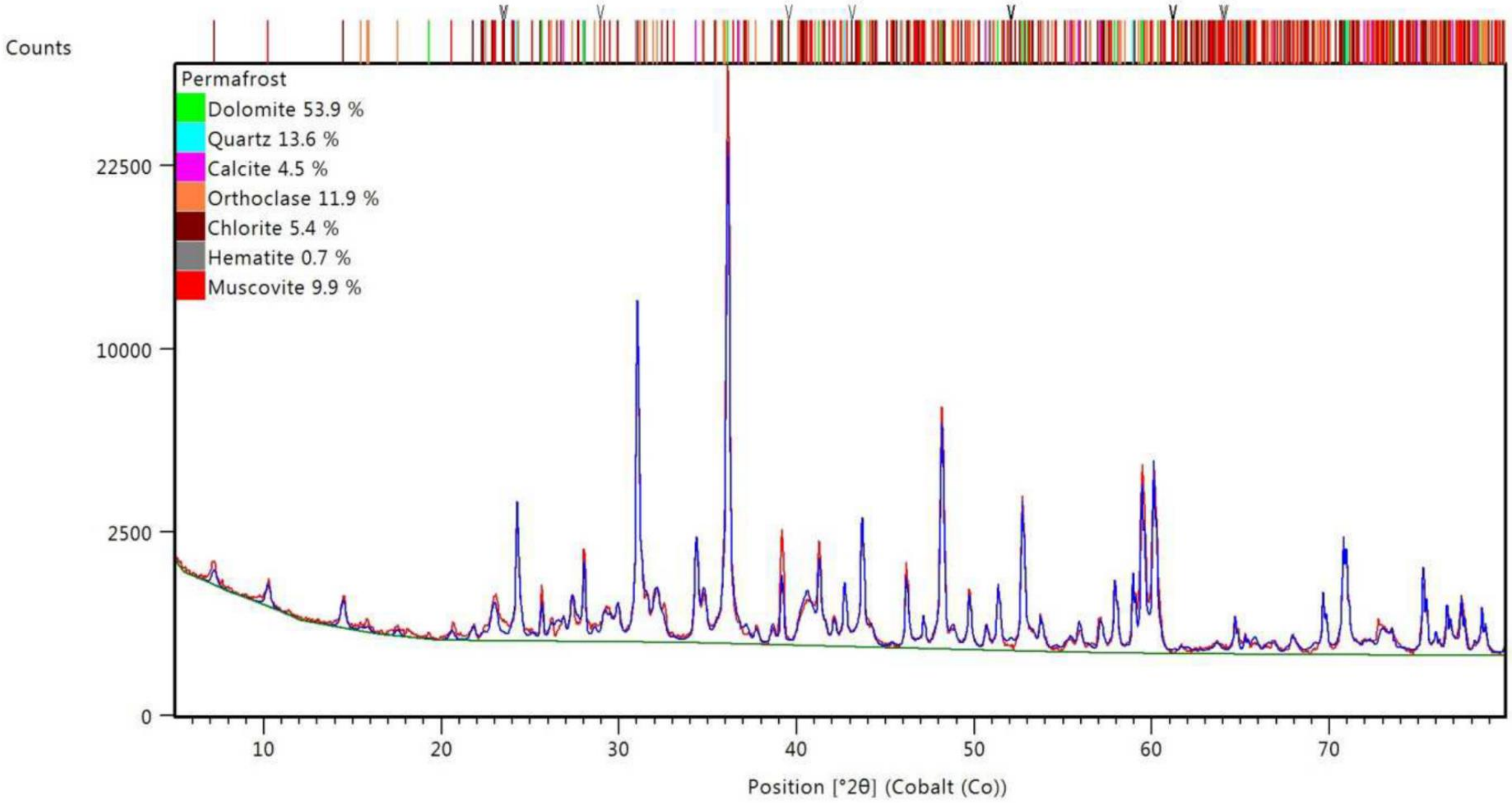

Supplementary Figure 2. Mineralogical analysis of the permafrost sample.

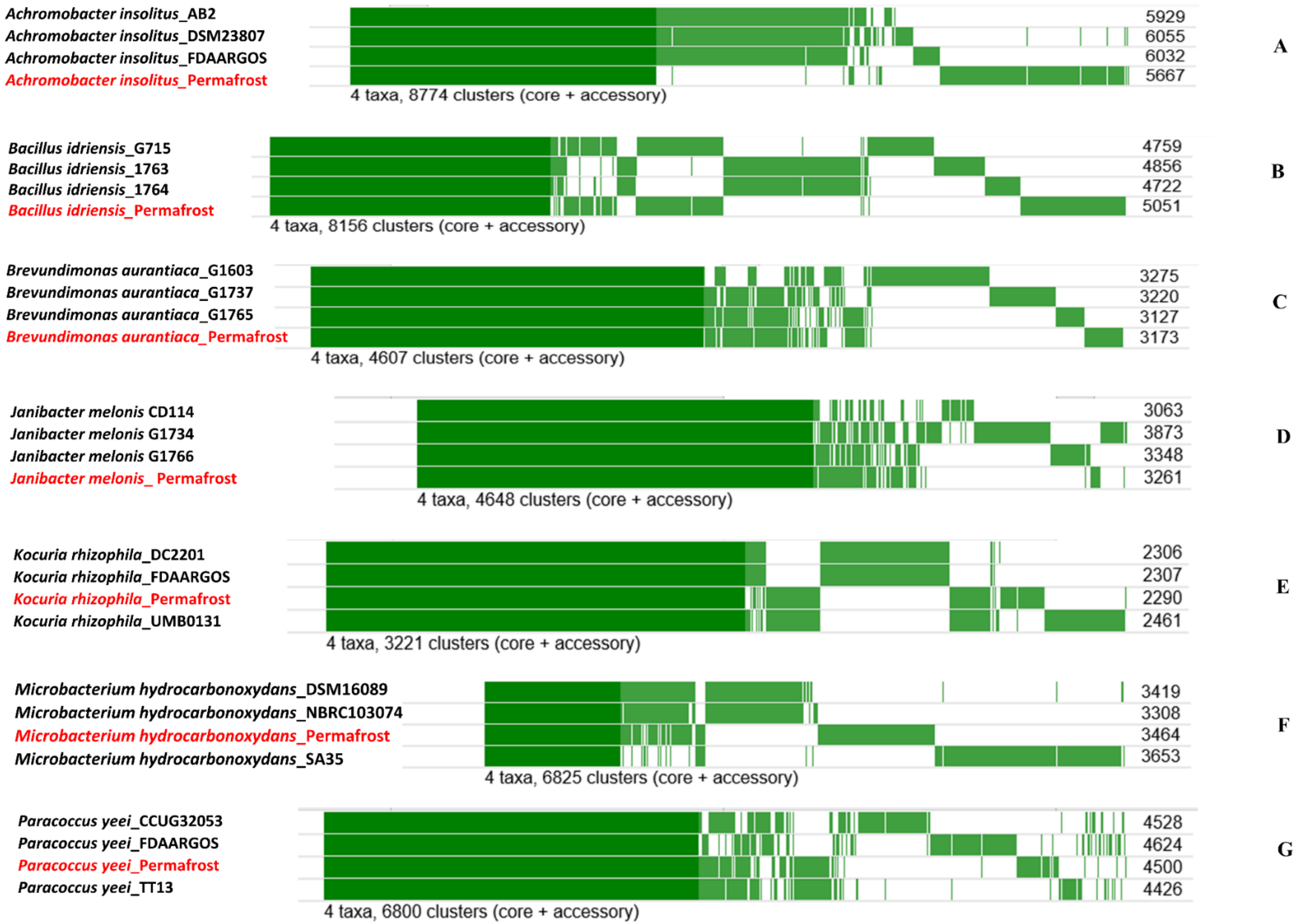

Supplementary Figure 3. Pan-genomes representing four strains of seven bacterial species: *Achromobacter insolitus* (A), *Bacillus idriensis* (B), *Brevundimonas aurantiaca* (C), *Janibacter melonis* (D), *Kocuria rhizophila* (E), *Microbacterium hydrocarbonoxydans* (F) and *Paracoccus yeei* (G). Isolated bacterial permafrost strains are in red and the other 3 strains represent the modern ones (non-permafrost). For each pan-genome the core is represented in dark green, and the genomic content related to the accessory genes and the unique genes are displayed in light green and white, respectively.

A

	AB2	DSM23807	FDAARGOS	Permafrost	Ancestral
AB2	-				
DSM23807	0.049	-			
FDAARGOS	0.053	0.052	-		
Permafrost	79.902	79.889	79.905	-	
Ancestral	0.031	0.018	0.034	79.871	-

Achromobacter insolitus

B

	G1763	G1764	G715	Permafrost	Ancestral
G1763	-				
G1764	2.465	-			
G715	5.266	5.268	-		
Permafrost	5.268	5.27	2.848	-	
Ancestral	2.45	2.452	2.816	2.818	-

Bacillus idriensis

C

	G1603	G1737	G1765	Permafrost	Ancestral
G1603	-				
G1737	0.906	-			
G1765	1.6	1.421	-		
Permafrost	1.628	1.449	1.187	-	
Ancestral	0.784	0.605	0.816	0.844	-

Brevundimonas aurantiaca

D

	CD114	G1734	G1766	Permafrost	Ancestral
CD114	-				
G1734	1.212	-			
G1766	1.022	0.864	-		
Permafrost	1.16	1.002	0.812	-	
Ancestral	0.605	0.607	0.417	0.555	-

Janibacter melonis

E

	DC2201	FDAARGOS	UMB0131	Permafrost	Ancestral
DC2201	-				
FDAARGOS	10.001	-			
UMB0131	20.953	20.953	-		
Permafrost	21.208	21.208	11.207	-	
Ancestral	10.001	10.001	10.952	11.207	-

Kocuria rhizophila

F

	DSM16089	NBRC103074	SA35	Permafrost	Ancestral
DSM16089	-				
NBRC103074	3.14	-			
SA35	3.211	3.211	-		
Permafrost	26.145	26.145	23.076	-	
Ancestral	3.14	3.14	0.071	23.005	-

Microbacterium hydrocarbonoxydans

G

	CCUG32053	FDAARGOS	TT13	Permafrost	Ancestral
CCUG32053	-				
FDAARGOS	2.621	-			
TT13	2.633	3.569	-		
Permafrost	2.642	3.578	1.121	-	
Ancestral	0.163	2.458	2.47	2.479	-

Paracoccus yeei

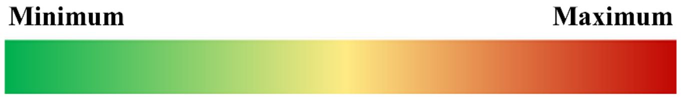

Supplementary Figure 4. Evolutionary distances of bacterial sequences relative to each other and relative to their ancestral sequences. The evolutionary distances are based on the length of branches of phylogenetic trees; these lengths were computed using the Maximum Composite Likelihood method [33] and are in units of the number of base substitutions per site. Green to red shows the lowest to highest SNPs. The column named ancestral represents ancestral sequences reconstructed with degenerated nucleotides by a python script. The column named "Permafrost" represents bacterial sequences from bacteria isolated from

permafrost; the others represent the modern non-permafrost sequences. *Achromobacter insolitus* (A), *Bacillus idriensis* (B), *Brevundimonas aurantiaca* (C), *Janibacter melonis* (D), *Kocuria rhizophila* (E), *Microbacterium hydrocarbonoxydans* (F) and *Paracoccus yeei* (G).

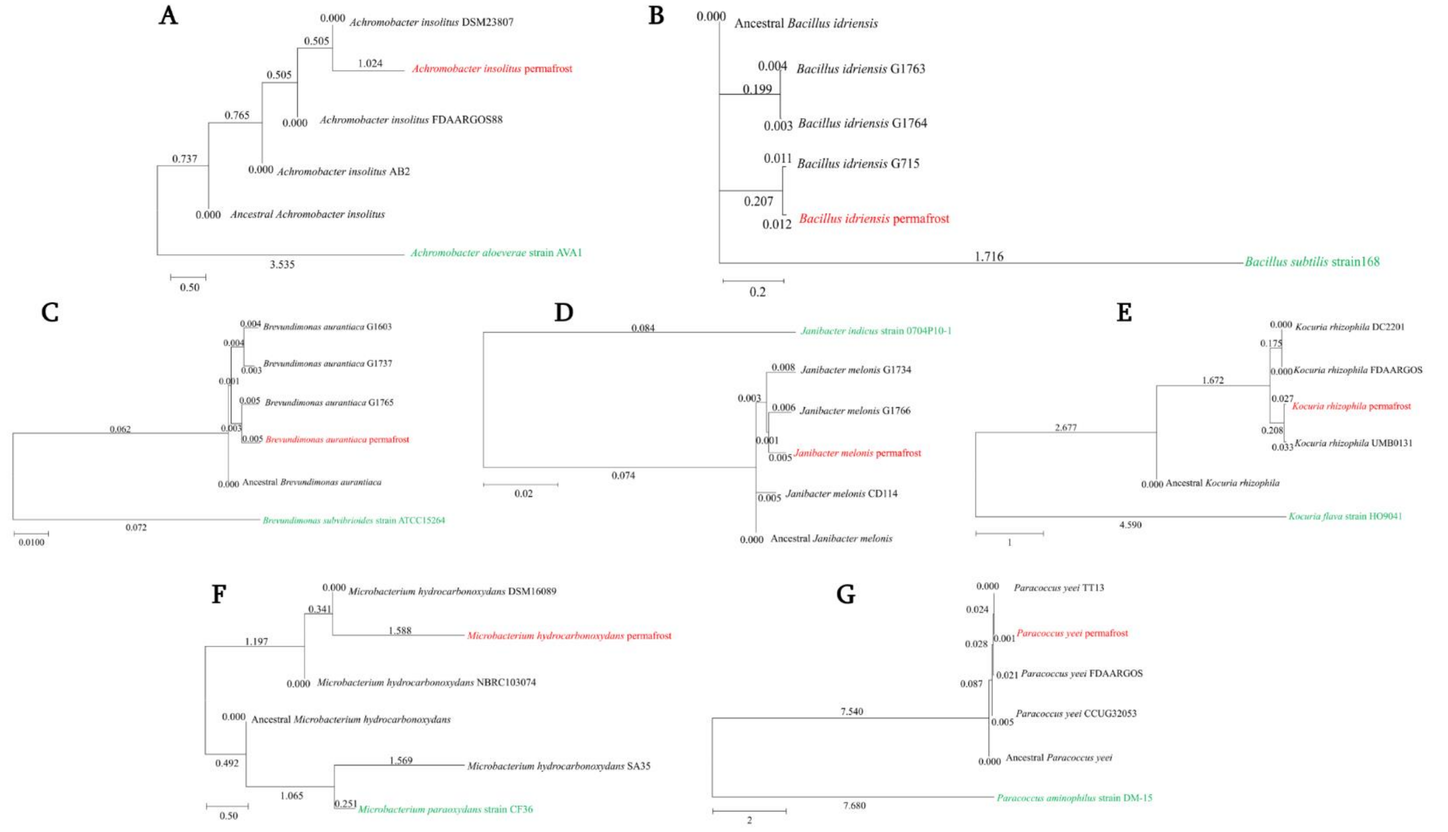

Supplementary Figure 5. Phylogenetic trees based on the SNP core genomes highlighting the position of permafrost species (set in red) compared to their modern equivalents and their ancestral sequences. The nearest cousin bacterial species (set in green) is used as an out-group. (A) *Achromobacter insolitus*, (B) *Bacillus idriensis*, (C) *Brevundimonas aurantiaca*, (D) *Janibacter melonis*, (E) *Kocuria rhizophila*, (F) *Microbacterium hydrocarbonoxydans* and (G) *Paracoccus yeei.* The evolutionary history was inferred using the Neighbor-Joining method [34]. The tree is drawn to scale, with branch lengths (next to the branches) in the same units as those of the evolutionary distances used to infer the phylogenetic tree. The evolutionary distances were computed using the Maximum Composite Likelihood method [33] and are in the units of the number of base substitutions per site. The analysis involved 6 nucleotide sequences. All positions containing gaps and missing data were eliminated. Evolutionary analyses were conducted in MEGAX [26].

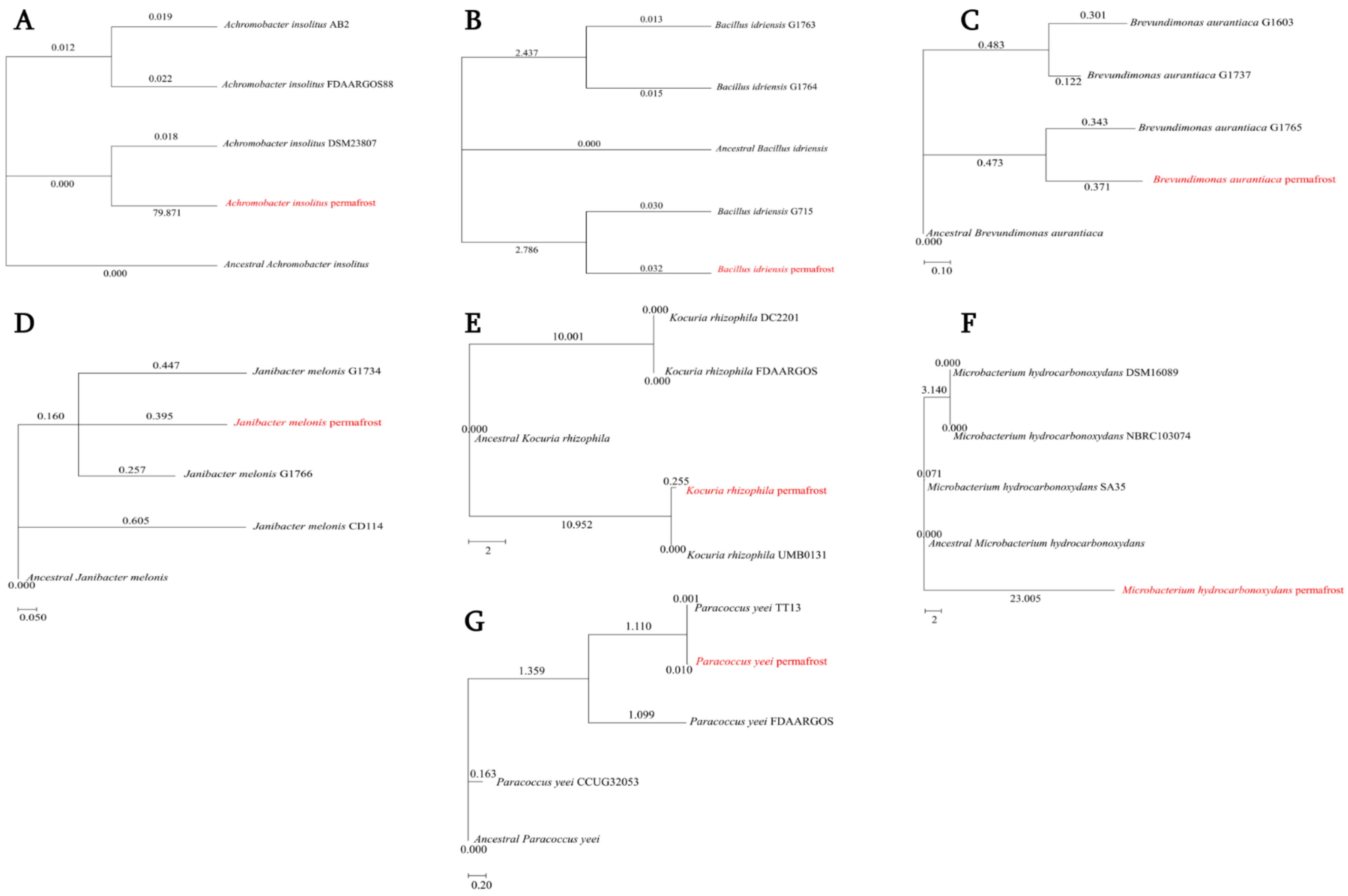

Supplementary Figure 6. Phylogenetic trees based on the SNP core genomes highlighting the position of strains of the same species compared to their ancestral sequences. Permafrost strains are displayed in red. (A) *Achromobacter insolitus* strains, (B) *Bacillus idriensis* strains, (C) *Brevundimonas aurantiaca* strains, (D) *Janibacter melonis* strains, (E) *Kocuria rhizophila* strains, (F) *Microbacterium hydrocarbonoxydans* strains and (G) *Paracoccus yeei* strains. The evolutionary history was inferred using the Neighbor-Joining method [34]. The tree is drawn to scale, with branch lengths (next to the branches) in the same units as those of the evolutionary distances used to infer the phylogenetic tree. The evolutionary distances were computed using the Maximum Composite Likelihood method [33]

and are in the units of the number of base substitutions per site. The analysis involved 6 nucleotide sequences. All positions containing gaps and missing data were eliminated. Evolutionary analyses were conducted in MEGAX [26].

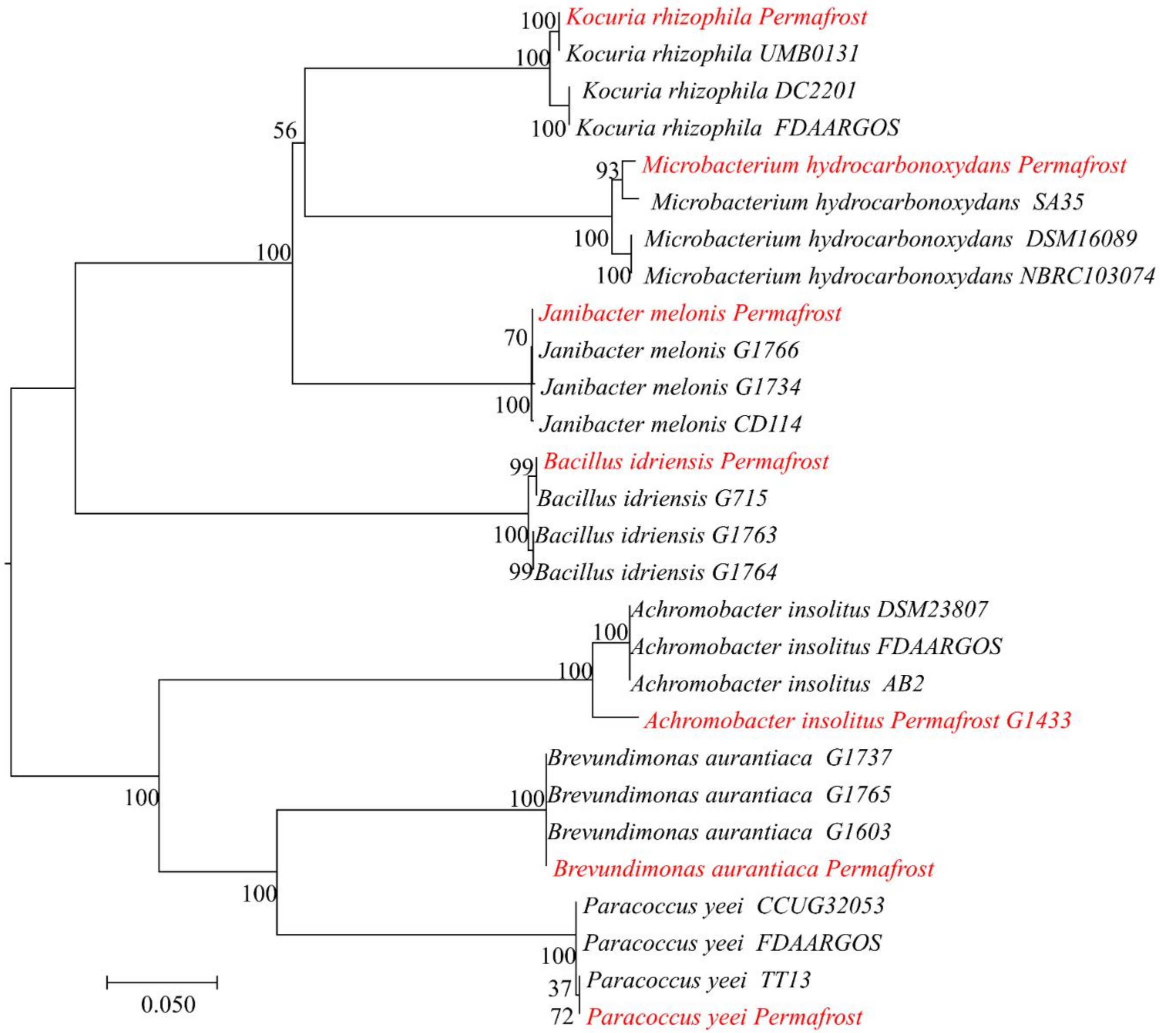

<u>Supplementary Figure 7.</u> Phylogenetic trees based on rpoB protein sequences of seven strains isolated from permafrost (in red) (*Achromobacter insolitus*, *Bacillus idriensis*, *Brevundimonas aurantiaca*, *Janibacter melonis*, *Kocuria rhizophila*, *Microbacterium hydrocarbonoxydans*, *Paracoccus yeei*) and three of the modern strains (in black) for each species. The evolutionary history was inferred using the Neighbor-Joining method. The optimal tree with the sum of branch length = 1.35628898 is shown. The percentage of replicate trees in which the associated taxa clustered together in the bootstrap test (1000 replicates) are shown next to the branches. The tree is drawn to scale, with branch lengths in the same units as those of the evolutionary distances used to infer the phylogenetic tree. The evolutionary distances were computed using the Poisson correction method and are in the units of the number of amino acid substitutions per site. The analysis involved 28 amino acid sequences. All positions containing gaps and missing data were eliminated. There were a total of 869 positions in the final dataset. Evolutionary analyses were conducted in MEGA7.

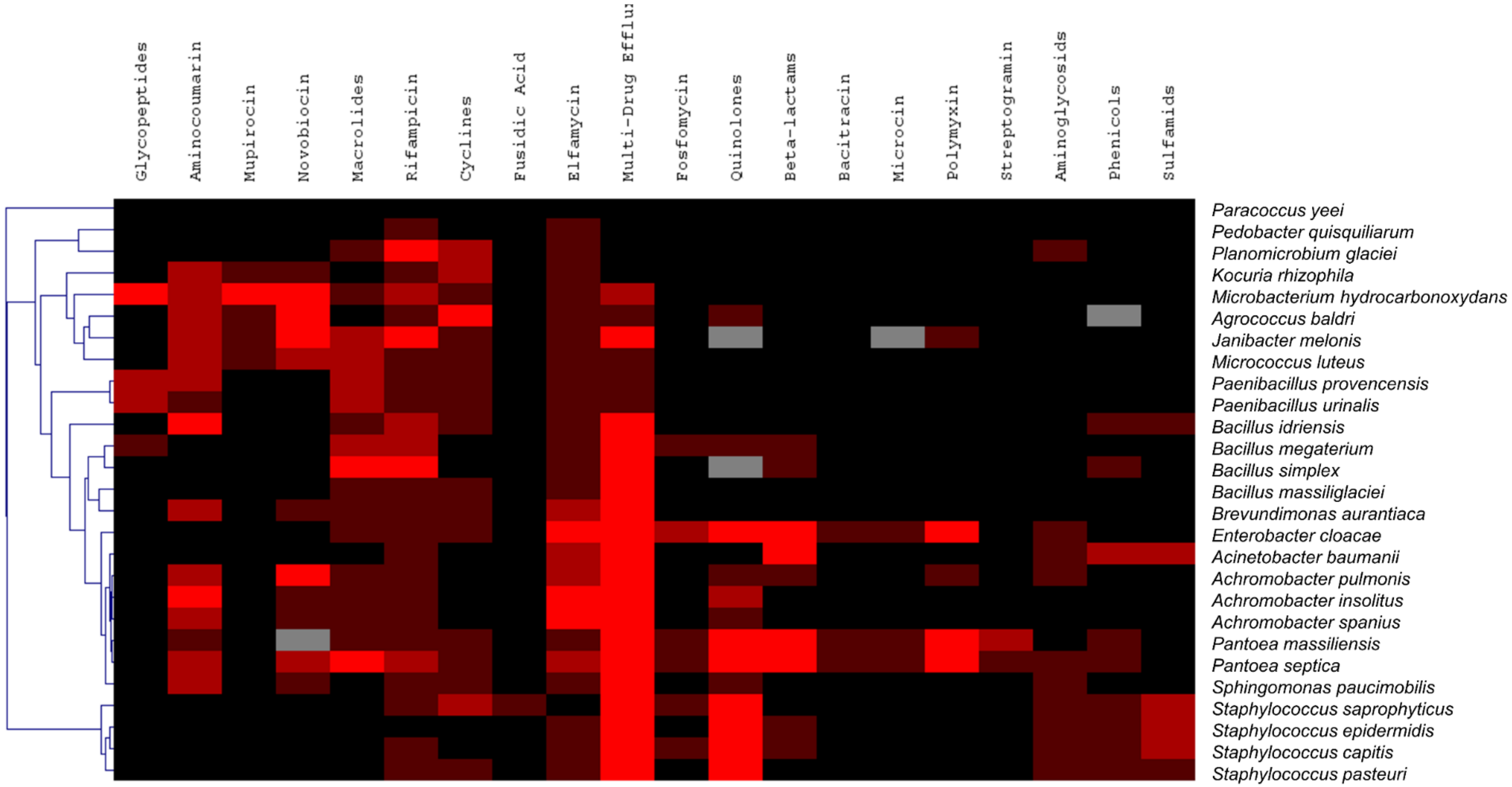

Supplementary Figure 8. Resistome of permafrost strains genome: BlastP analysis against ARG-ANNOT, CARD, and Resfinder Databases.
